# Supplementary material for: Individual differences in wellbeing are supported by separable sets of co-active self- and visual-attention-related brain networks
Source: Sci Rep. 2025 Feb 14;15:5524. doi: 10.1038/s41598-025-86762-w (PMC11828889; doi:10.1038/s41598-025-86762-w)
Supplement: Supplementary file 1 — Supplementary Material 1 [file 41598_2025_86762_MOESM1_ESM.pdf]

Supplementary Information

Individual differences in wellbeing are supported by separable sets of co-active self- and visual-attention-related brain networks

Yumeng Ma[1,2,3,*] & Jeremy I Skipper[2]

[1] Department of Neuroscience, Physiology and Pharmacology, University College London, London, UK
[2] Experimental Psychology, University College London, London, UK
[3] Department of Psychology, Emory University, Atlanta, GA, USA
[*] Corresponding author: yumeng.ma@emory.edu

Figure S1. 50-dimensional spatial maps. *Spatial maps generated from independent component analysis with 50 components. Maps are thresholded to only include positive values and cluster sizes larger than 50 voxels, and any maps with no surviving clusters after thresholding are excluded from display. A red border highlights ICs with spatial maps that are most correlated with those of the 49 significant ICs identified from the 100-dimensional analysis in the Mantel test. The corresponding IC number and correlation value are shown.*

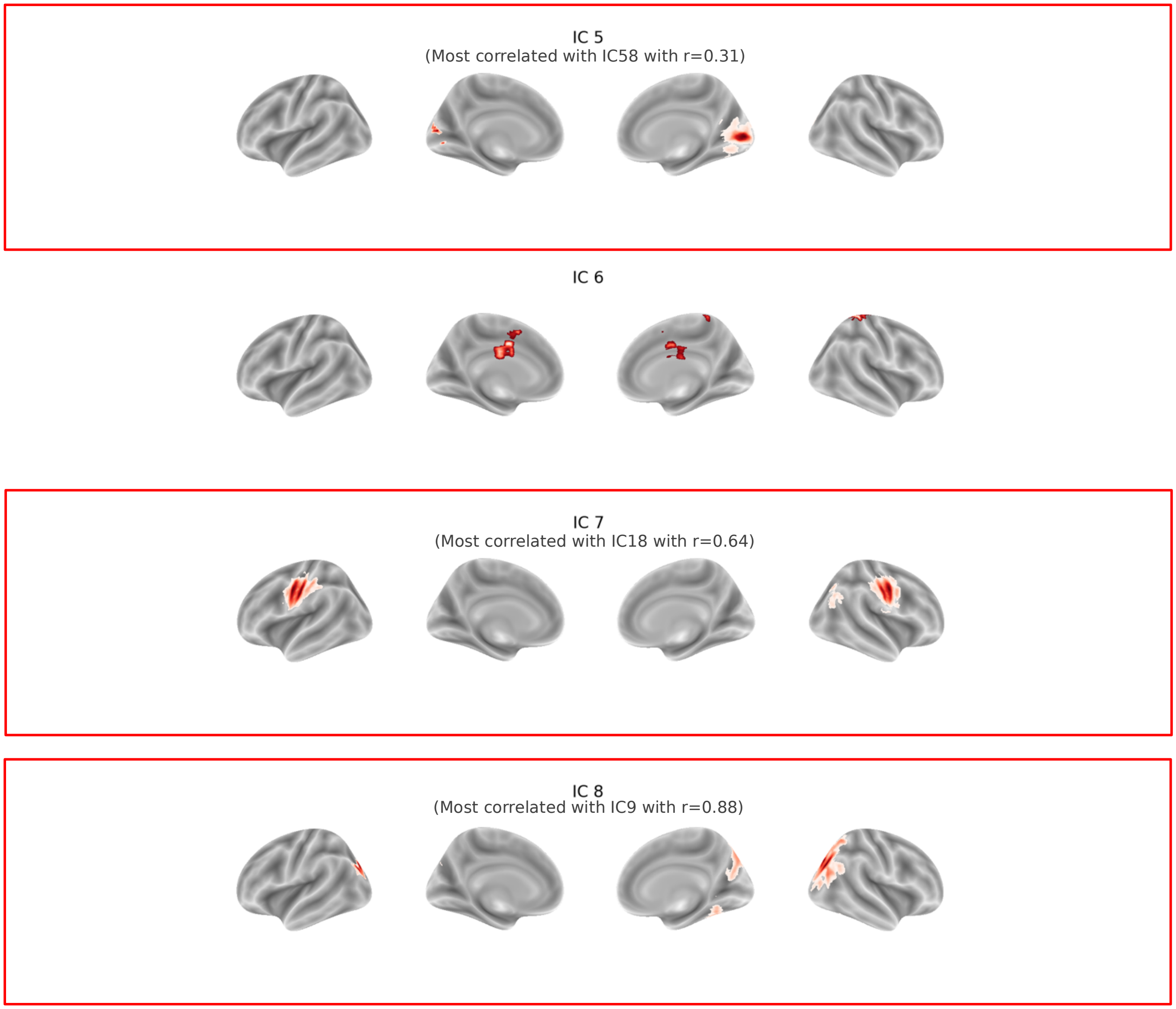

IC 9
(Most correlated with IC7 with r=0.93)

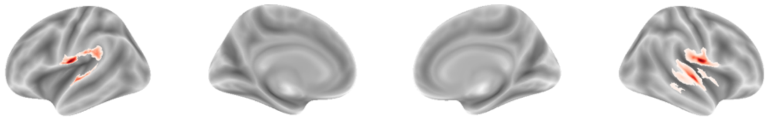

IC 10
(Most correlated with IC10 with r=0.92)

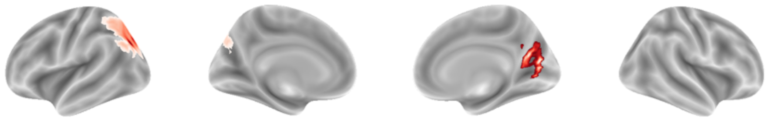

IC 11
(Most correlated with IC8 with r=0.94)

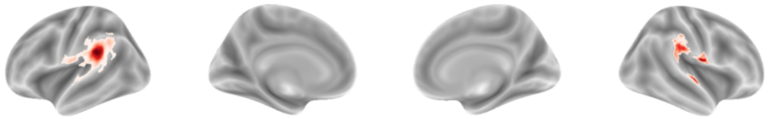

IC 12
(Most correlated with IC12 with r=0.83 and IC70 with r = 0.22)

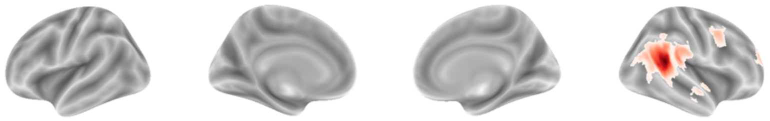

IC 13
(Most correlated with IC40 with r=0.39)

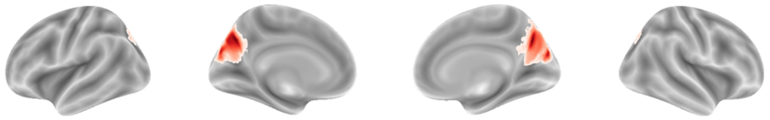

IC 14
(Most correlated with IC15 with r=0.87)

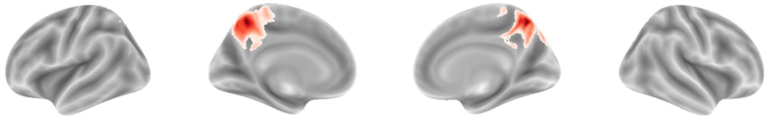

IC 15

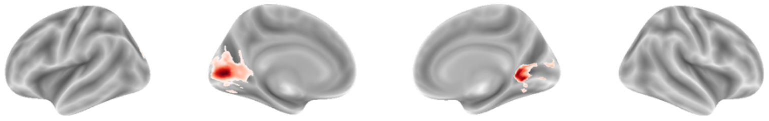

IC 16
(Most correlated with IC16 with r=0.89 and IC66 with r = 0.29)

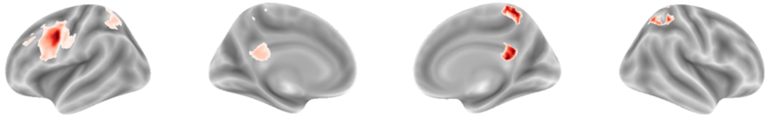

IC 17

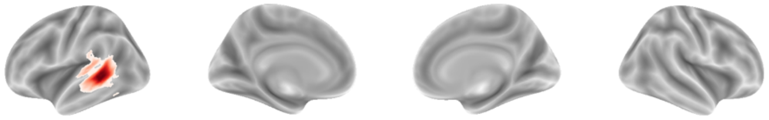

IC 18
(Most correlated with IC14 with r=0.80)

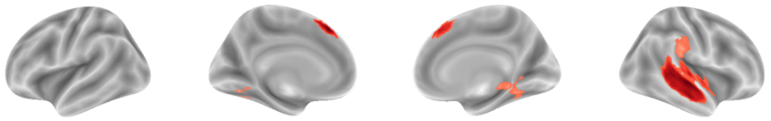

IC 19
(Most correlated with IC19 with r=0.88 and IC47 with r = 0.37)

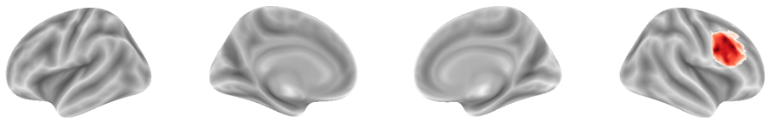

IC 20
(Most correlated with IC28 with r=0.80 and IC44 with r = 0.44)

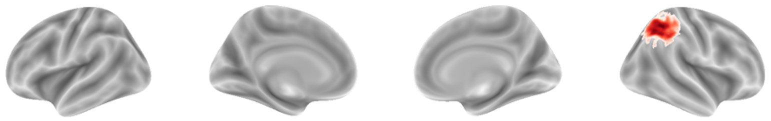

IC 21

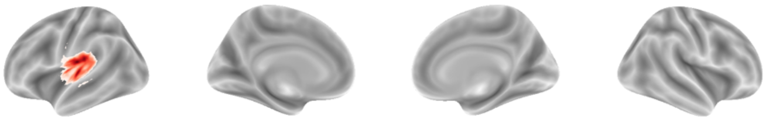

IC 22
(Most correlated with IC42 with r=0.73 and IC55 with r = 0.34)

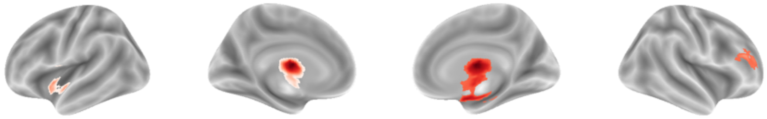

IC 23
(Most correlated with IC32 with r=0.79 and IC88 with r = 0.33)

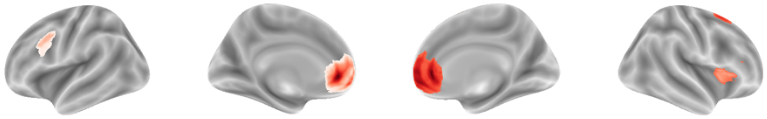

IC 24

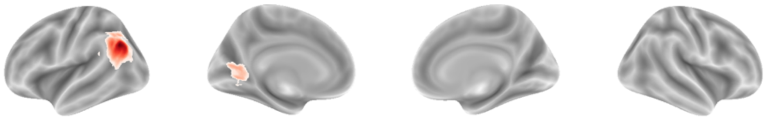

IC 25
(Most correlated with IC31 with r=0.57)

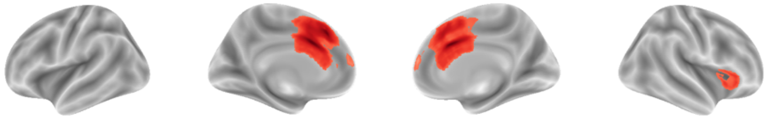

IC 26
(Most correlated with IC21 with r=0.66, IC90 with r = 0.21 and IC99 with r = 0.13)

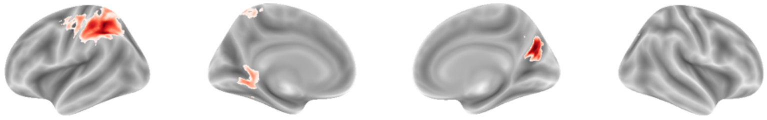

IC 27
(Most correlated with IC57 with r=0.25)

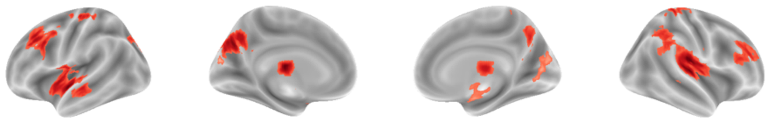

IC 28
(Most correlated with IC35 with r=0.60)

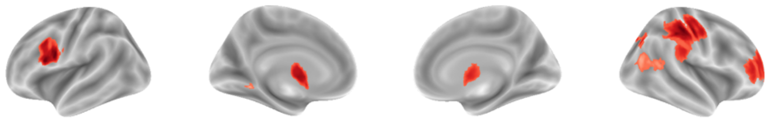

IC 29

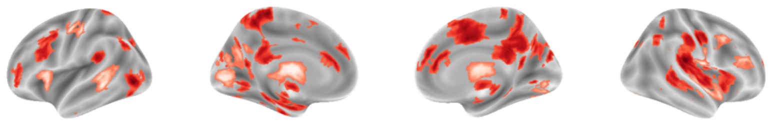

IC 30
(Most correlated with IC26 with r=0.76)

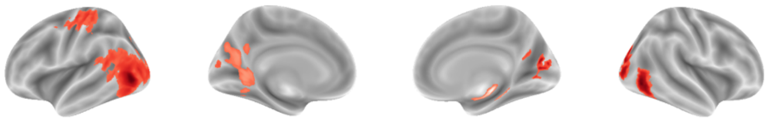

IC 31
(Most correlated with IC29 with r=0.56 and IC86 with r = 0.32)

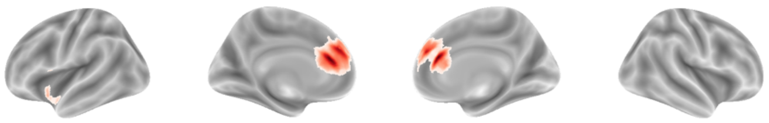

IC 32
(Most correlated with IC62 with r=0.17 and IC85 with r = 0.43)

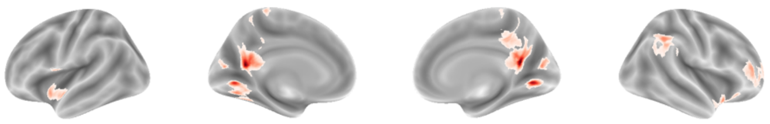

IC 33

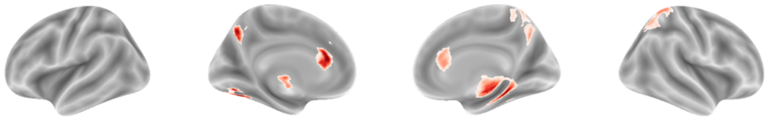

IC 34
(Most correlated with IC54 with r=0.65 and IC64 with r = 0.38)

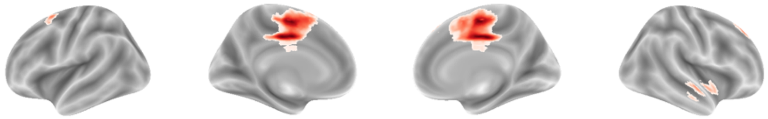

IC 35

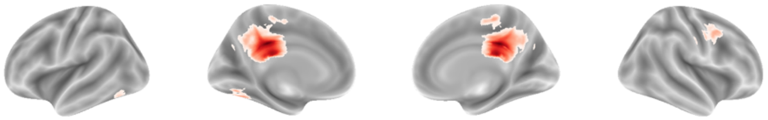

IC 36
(Most correlated with IC51 with r=0.69 and IC68 with r = 0.65)

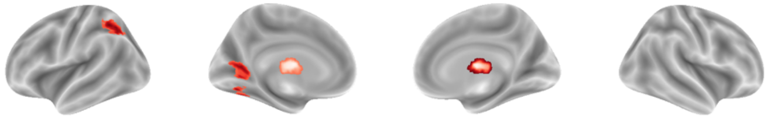

IC 37

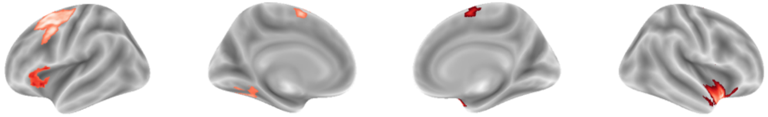

IC 38
(Most correlated with IC50 with r=0.73 and IC78 with r = 0.63)

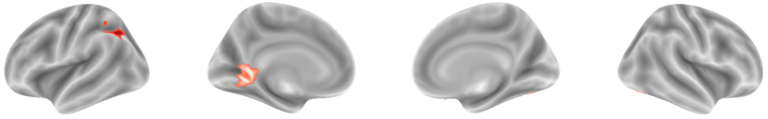

IC 39

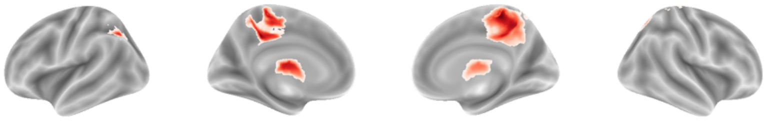

IC 40
(Most correlated with IC43 with r=0.79 and IC96 with r = 0.09)

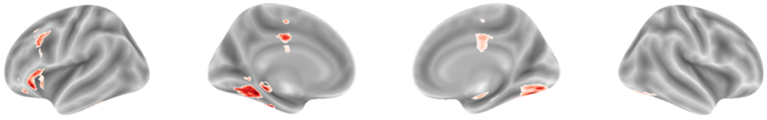

IC 41
(Most correlated with IC67 with r=0.17 and IC72 with r = 0.15)

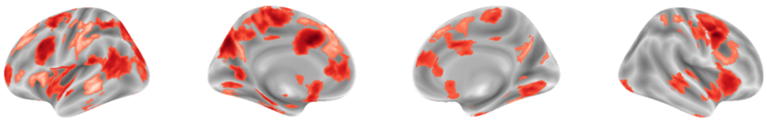

IC 42
(Most correlated with IC59 with r=0.48)

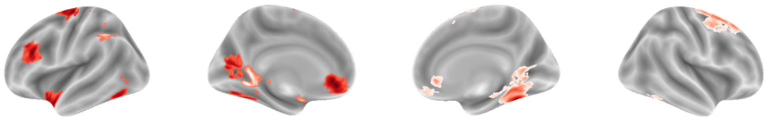

IC 43

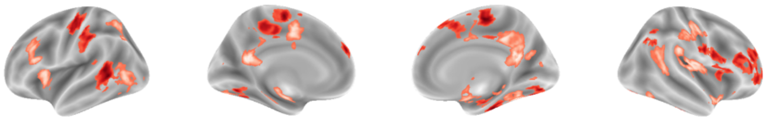

IC 44
(Most correlated with IC80 with r=0.24)

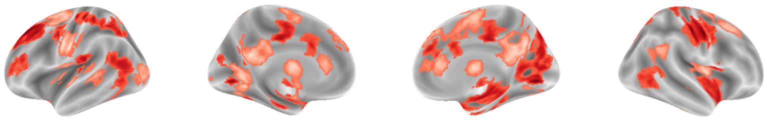

IC 45

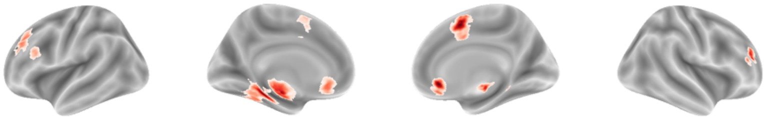

IC 46

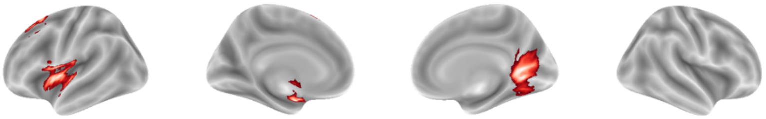

IC 47
(Most correlated with IC60 with r=0.70 and IC87 with r = 0.31)

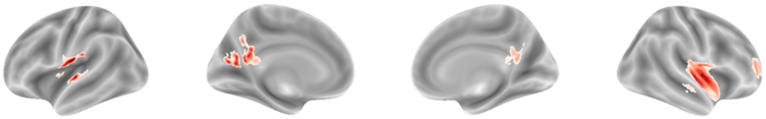

IC 48
(Most correlated with IC76 with r=0.36)

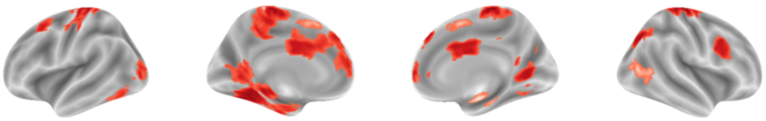

IC 49

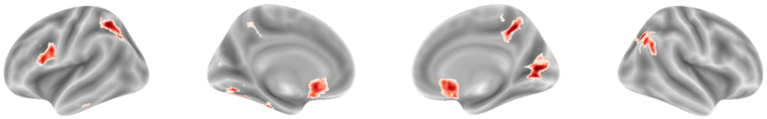

IC 50
(Most correlated with IC22 with r=0.16 and IC63 with r = 0.43)

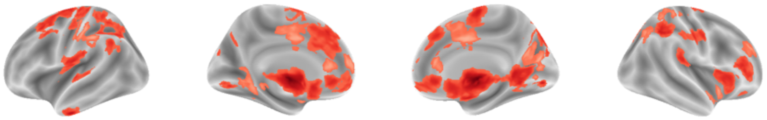

Figure S2. 100-dimensional spatial maps. *Spatial maps generated from independent component analysis with 100 components. Maps are thresholded to only include positive values and cluster sizes larger than 50 voxels, and any maps with no surviving clusters after thresholding are excluded from display.*

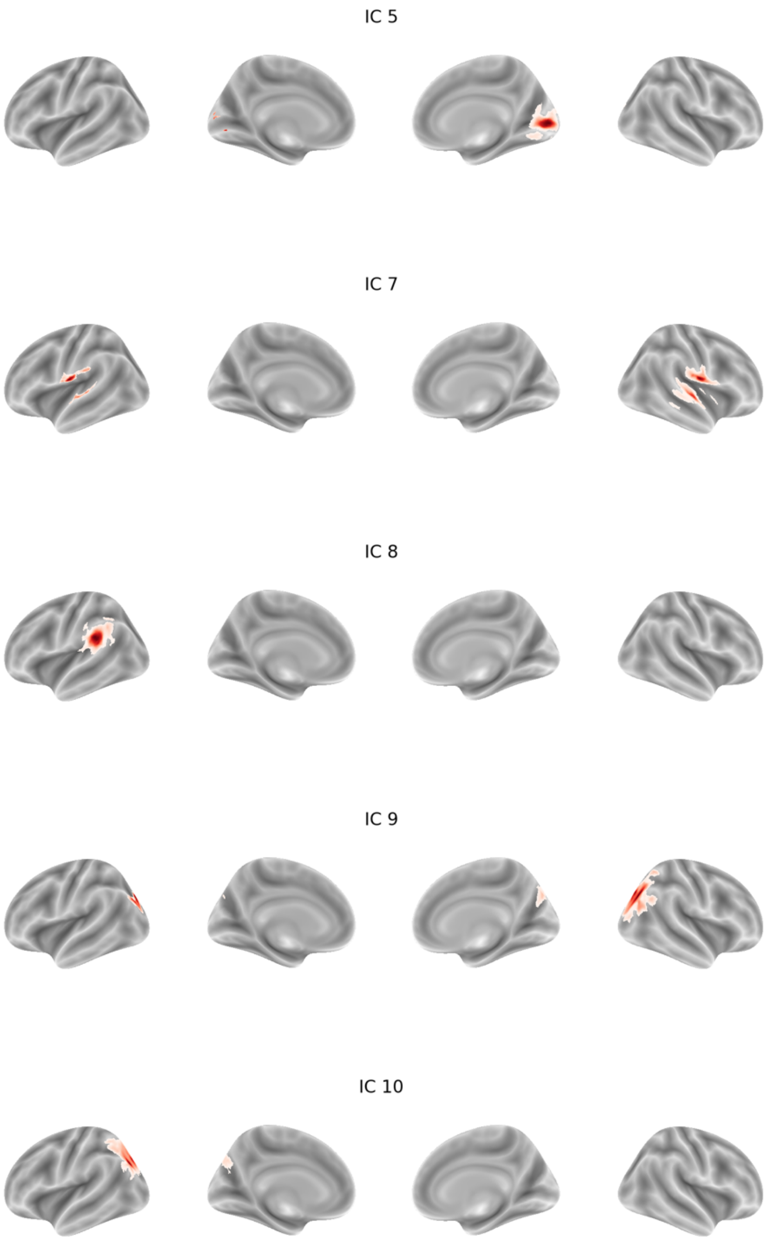

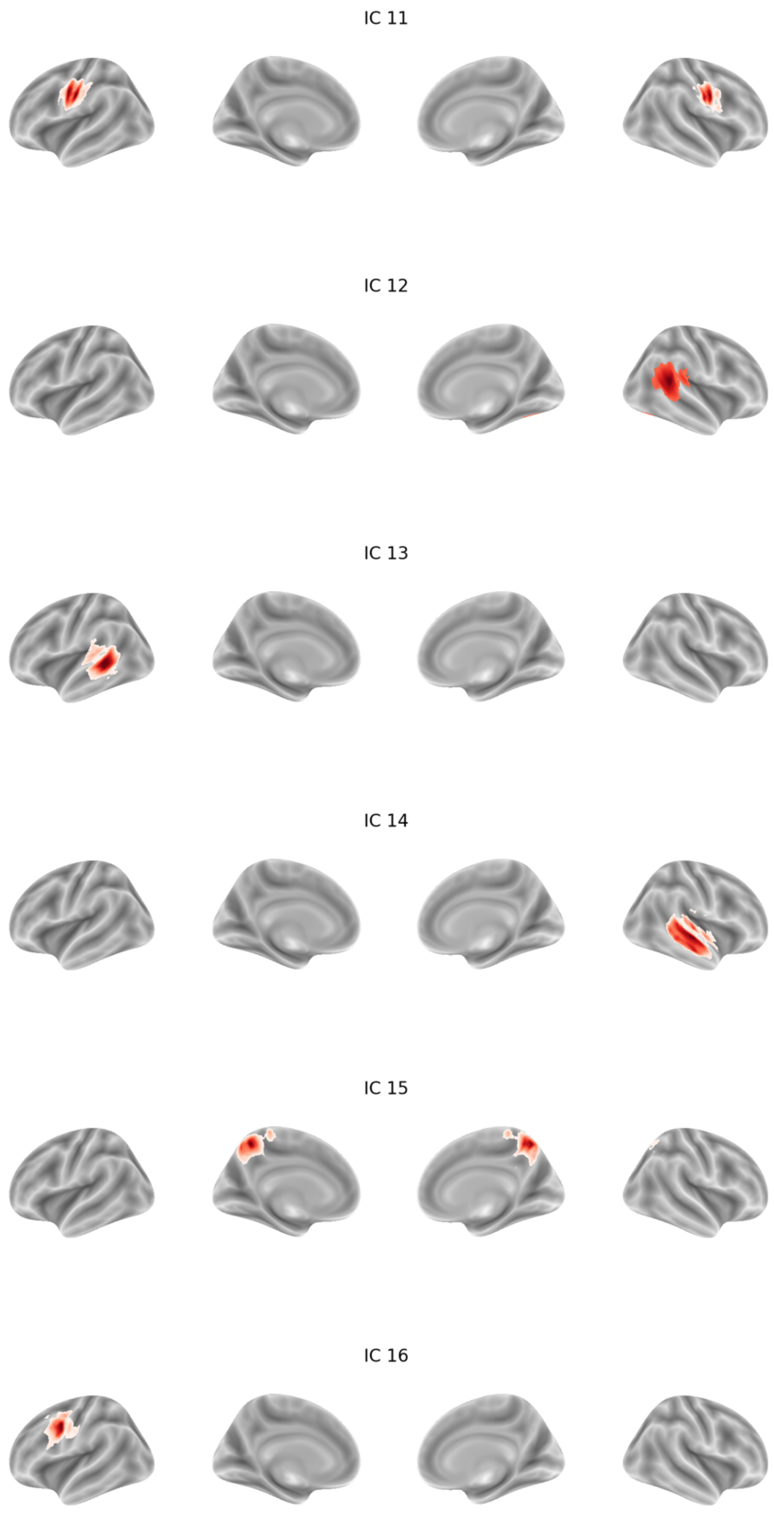
IC 11
IC 12
IC 13
IC 14
IC 15
IC 16

IC 17

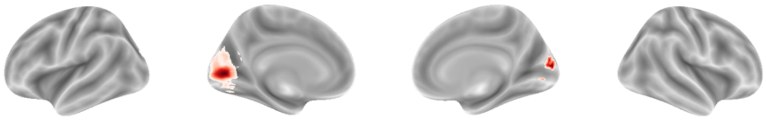

IC 18

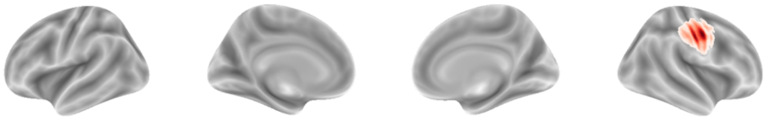

IC 19

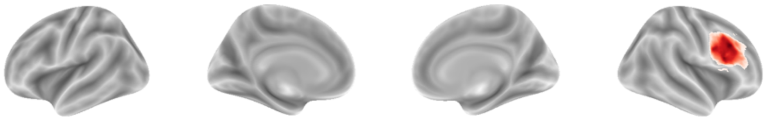

IC 20

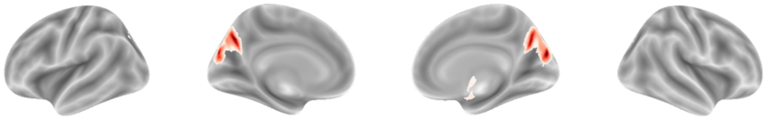

IC 21

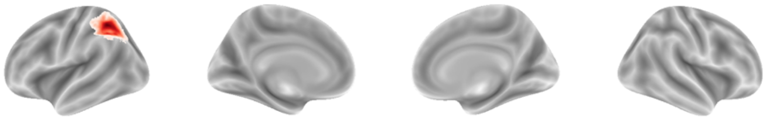

IC 22

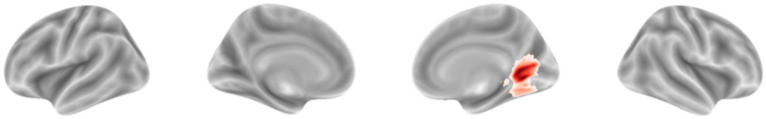

IC 23

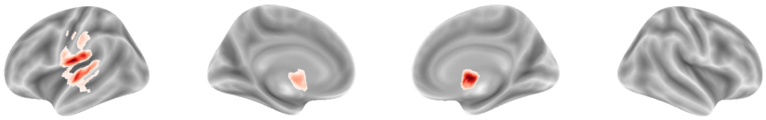

IC 24

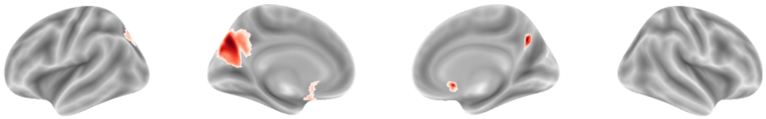

IC 25

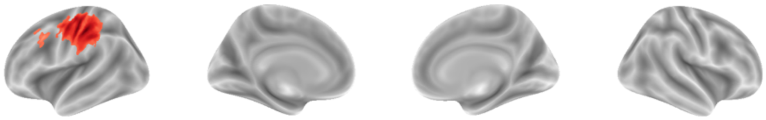

IC 26

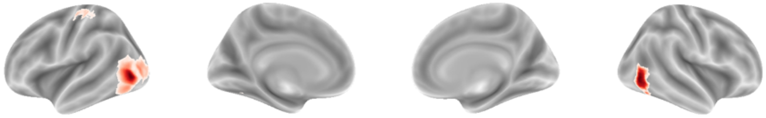

IC 27

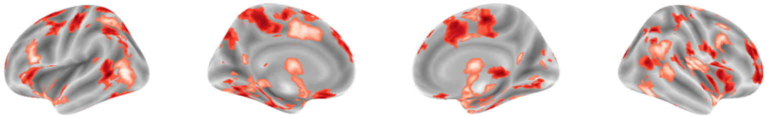

IC 28

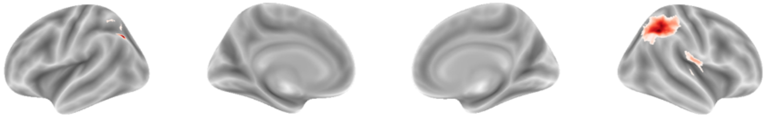

IC 29

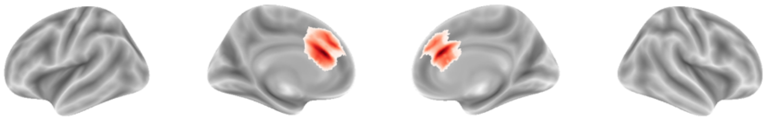

IC 30

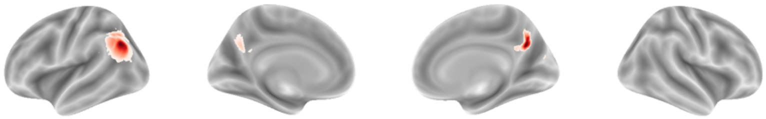

IC 31

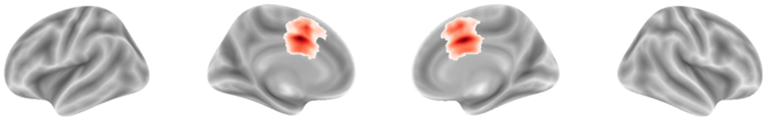

IC 32

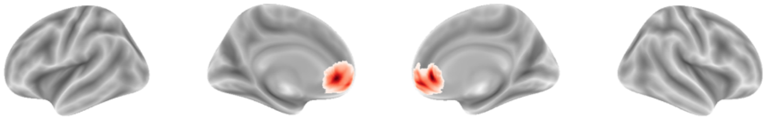

IC 33

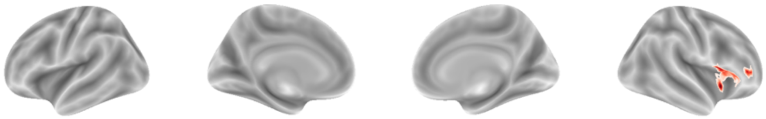

IC 34

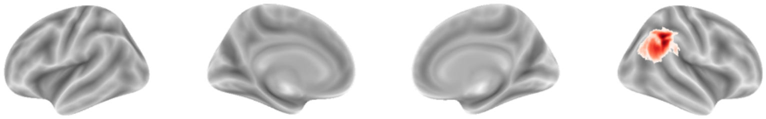

IC 35

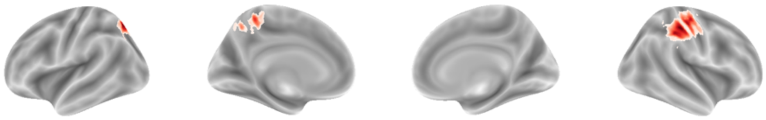

IC 36

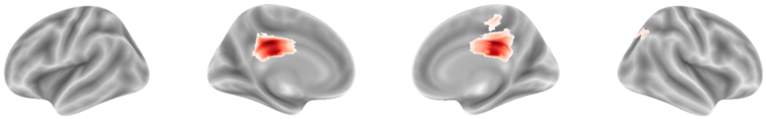

IC 37

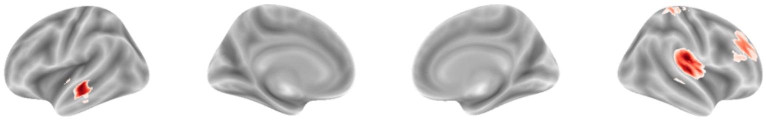

IC 38

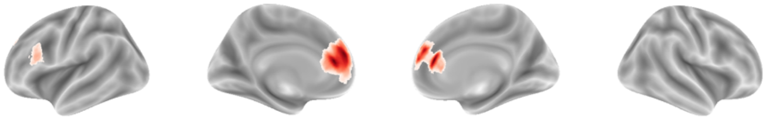

IC 39

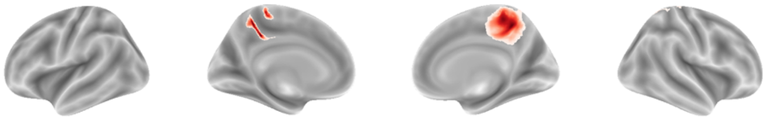

IC 40

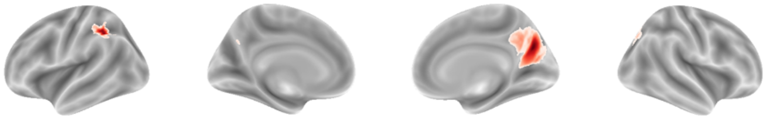

IC 41

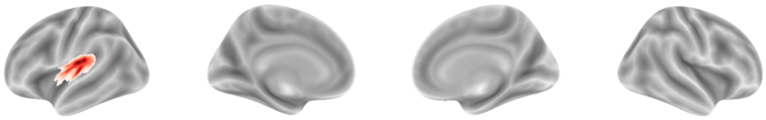

IC 42

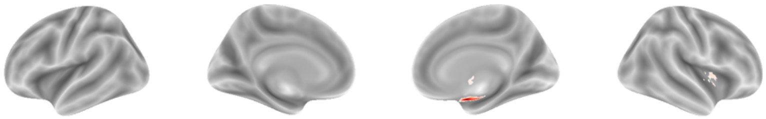

IC 43

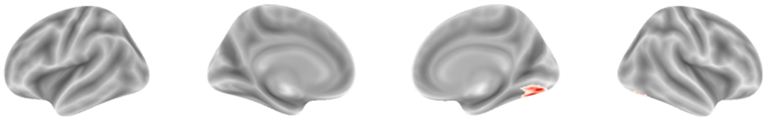

IC 44

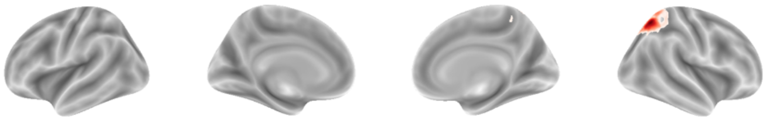

IC 45

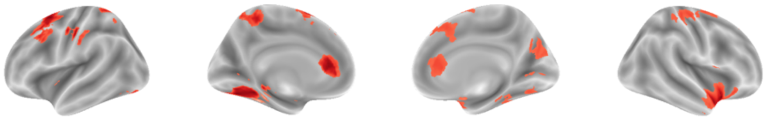

IC 46

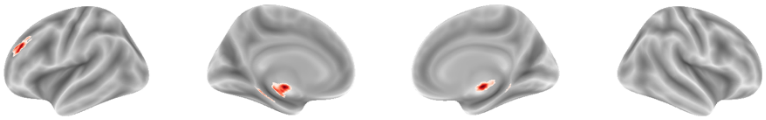

IC 47

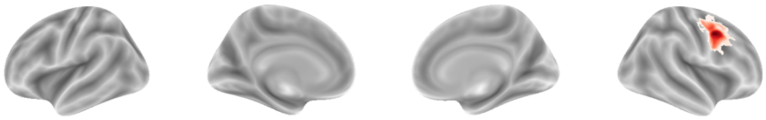

IC 48

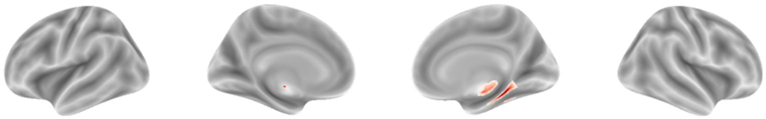

IC 49

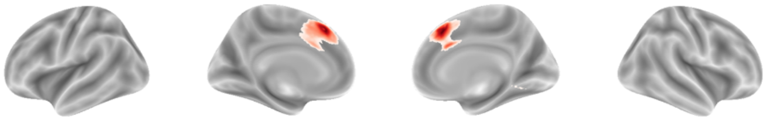

IC 50

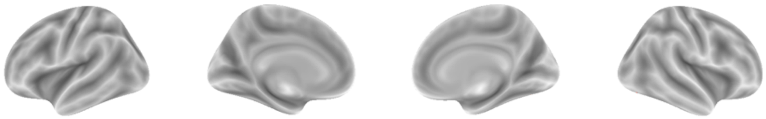

IC 51

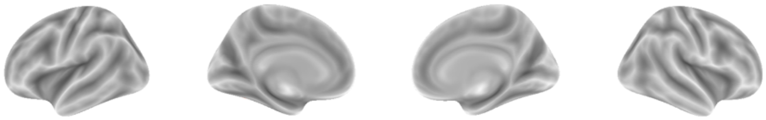

IC 52

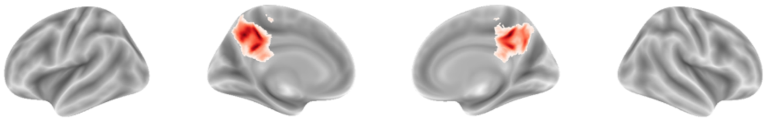

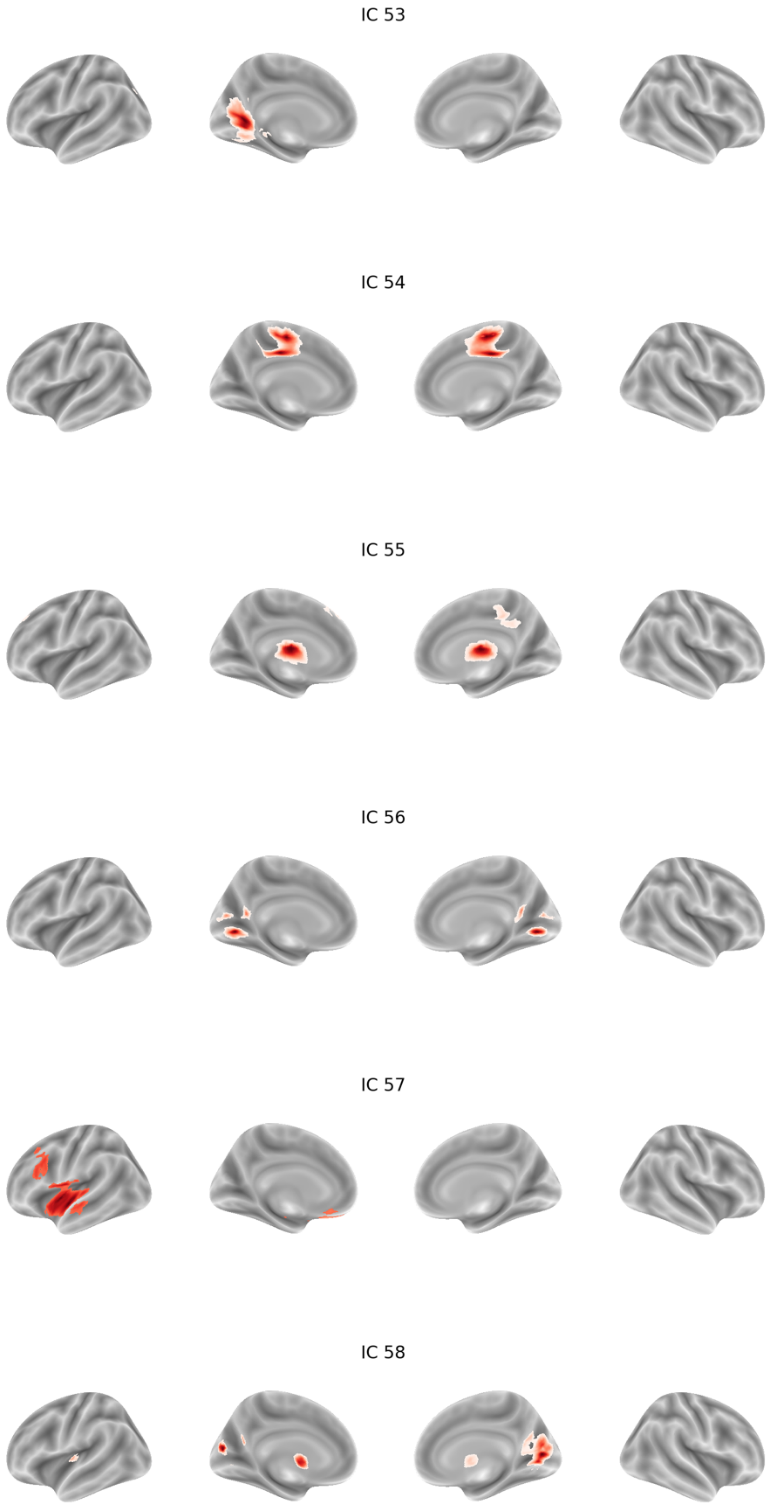
IC 53
IC 54
IC 55
IC 56
IC 57
IC 58

IC 59

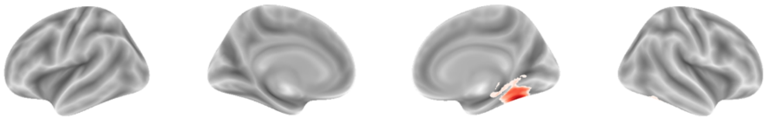

IC 60

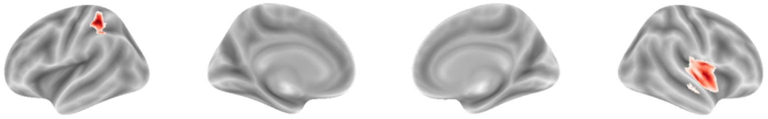

IC 61

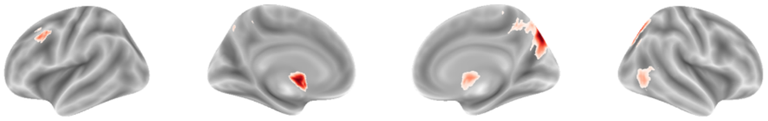

IC 62

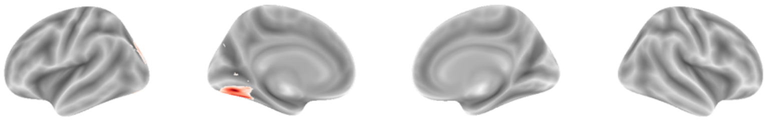

IC 63

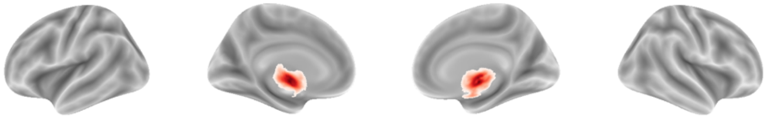

IC 64

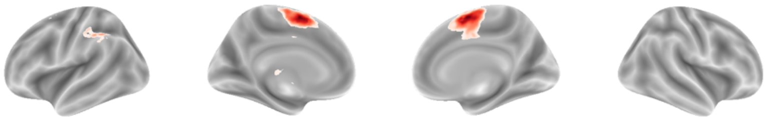

IC 65

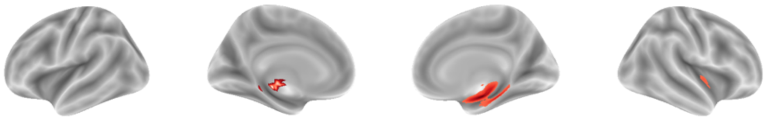

IC 66

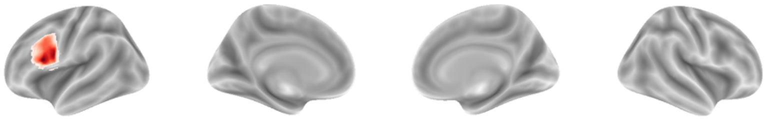

IC 67

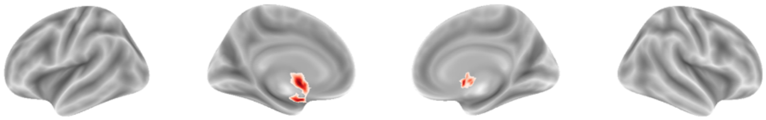

IC 68

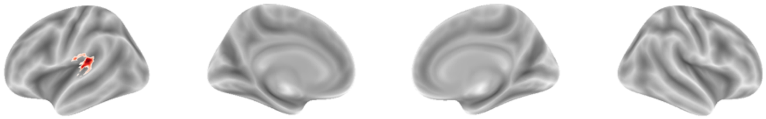

IC 69

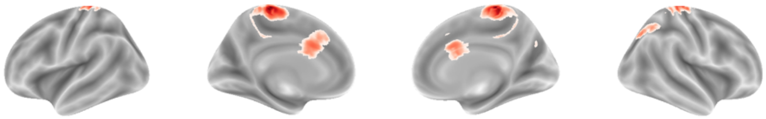

IC 70

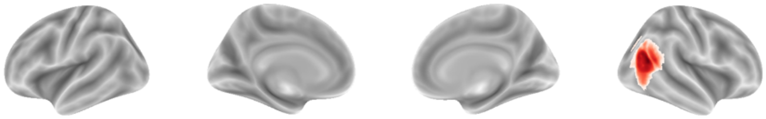

IC 71

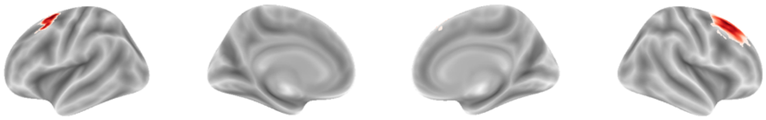

IC 72

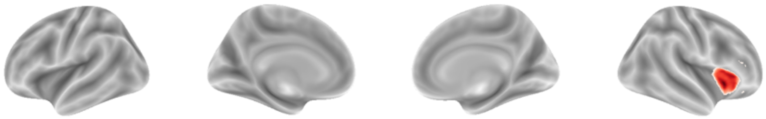

IC 73

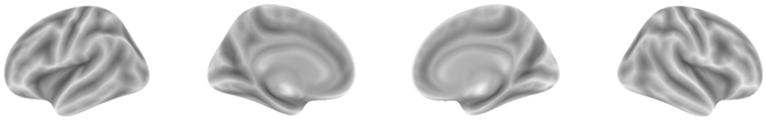

IC 74

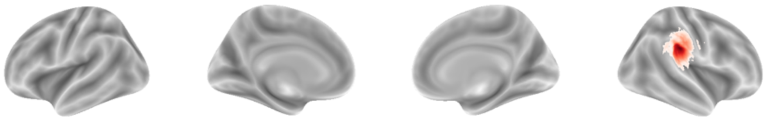

IC 75

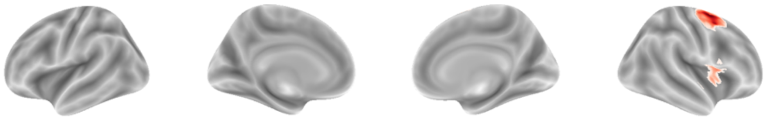

IC 76

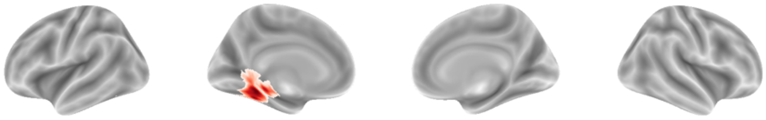

IC 77

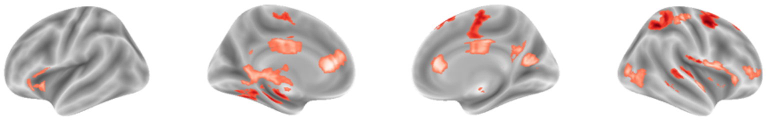

IC 78

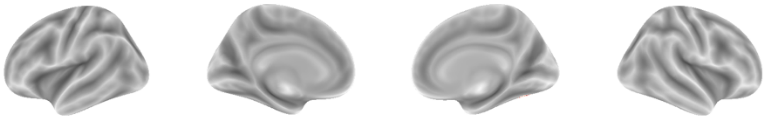

IC 79

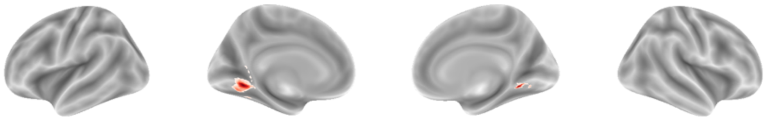

IC 80

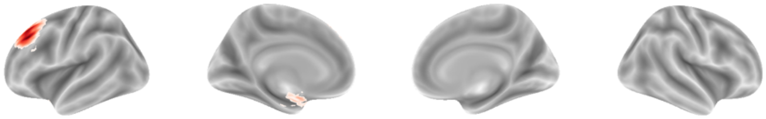

IC 81

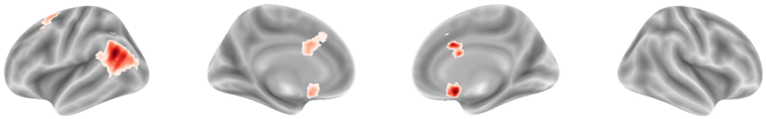

IC 82

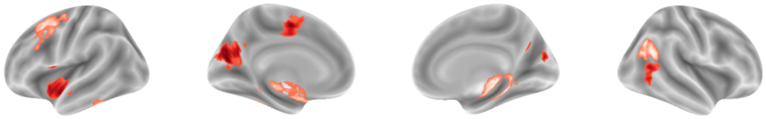

IC 83

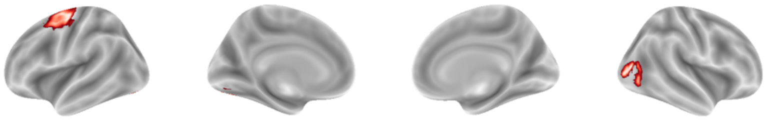

IC 84

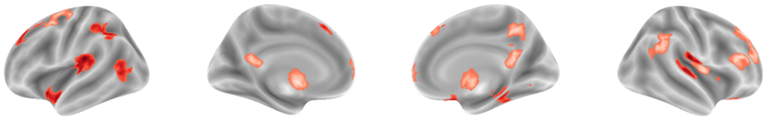

IC 85

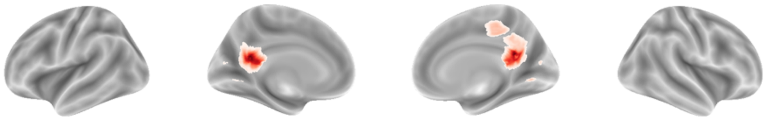

IC 86

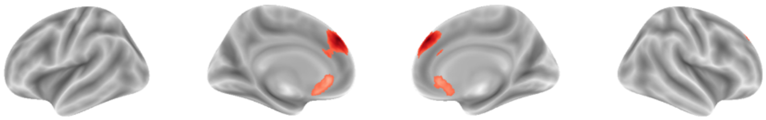

IC 87

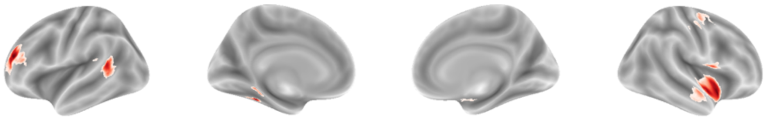

IC 88

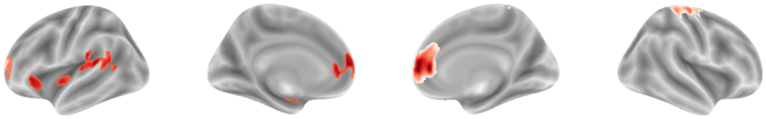

IC 89

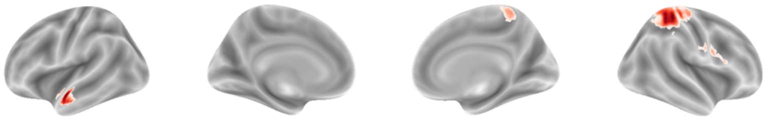

IC 90

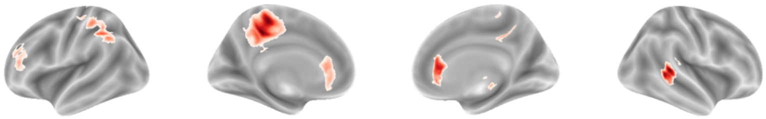

IC 91

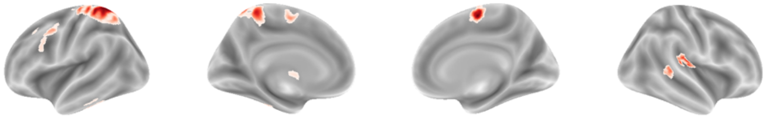

IC 92

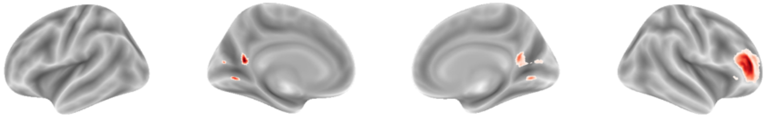

IC 93

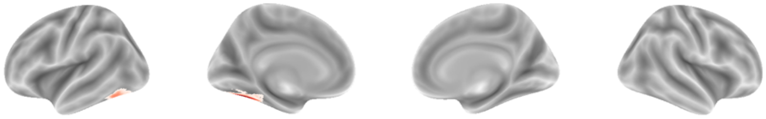

IC 94

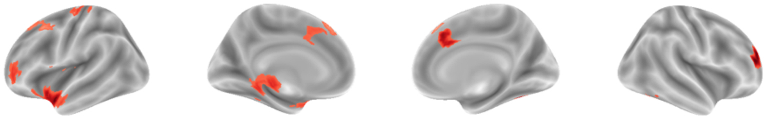

IC 95

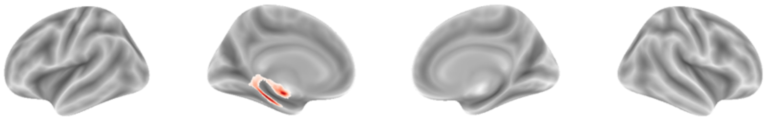

IC 96

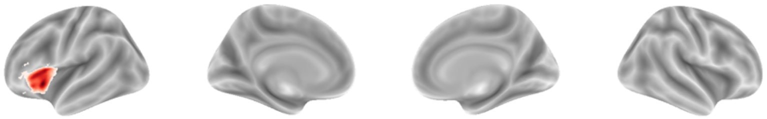

IC 97

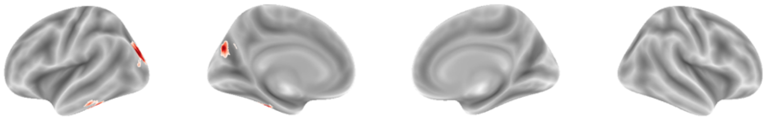

IC 98

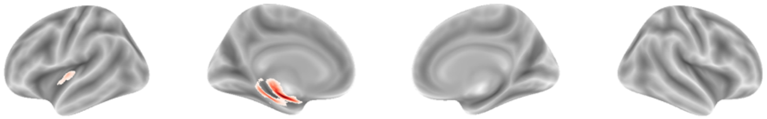

Figure S3. 150-dimensional spatial maps. *Spatial maps generated from independent component analysis with 150 components. Maps are thresholded to only include positive values and cluster sizes larger than 50 voxels, and any maps with no surviving clusters after thresholding are excluded from display. A red border highlights ICs with spatial maps that are most correlated with those of the 49 significant ICs identified from the 100-dimensional analysis in the Mantel test. The corresponding IC number and correlation value are shown.*

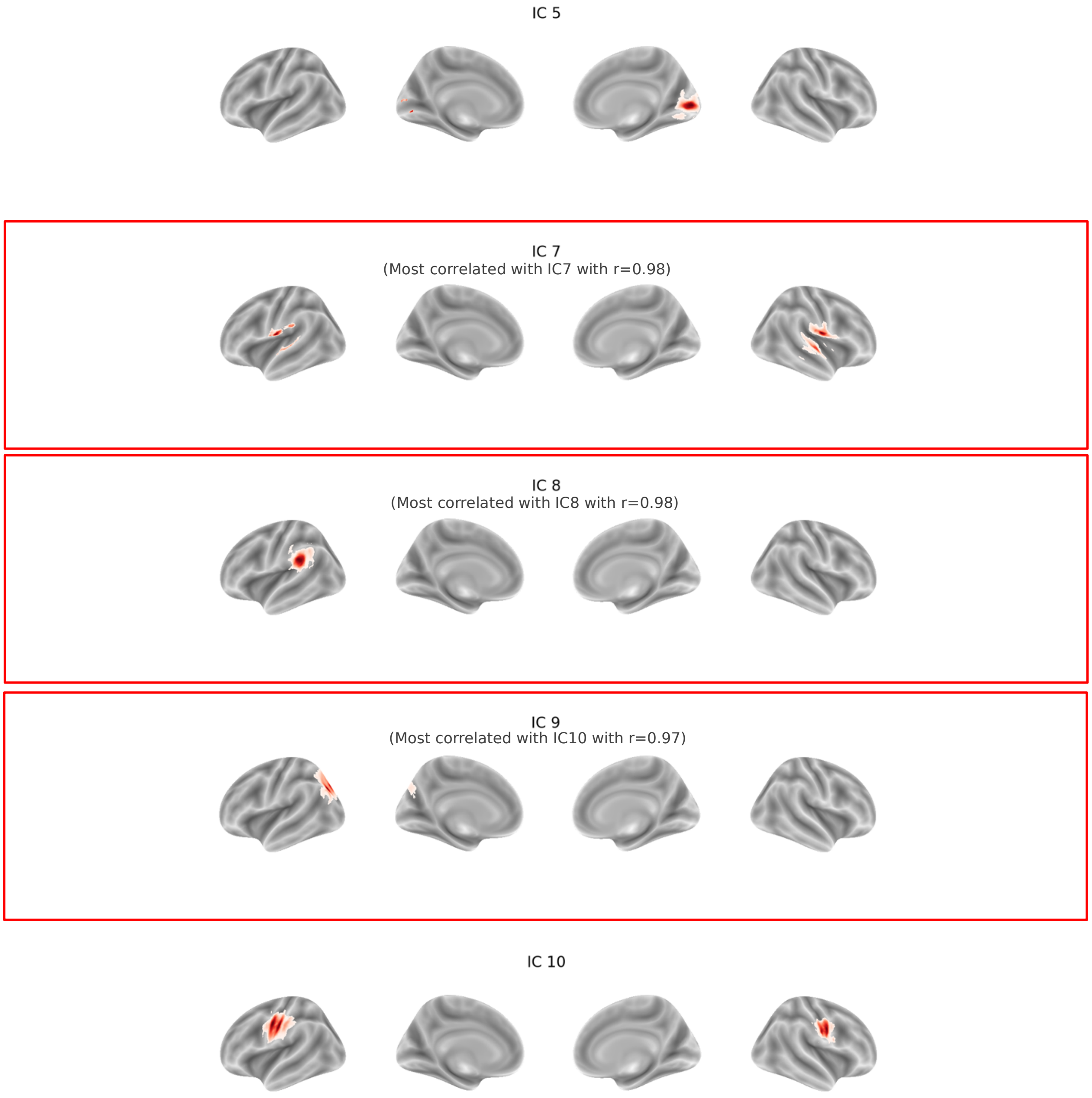

IC 11
(Most correlated with IC12 with r=0.95)

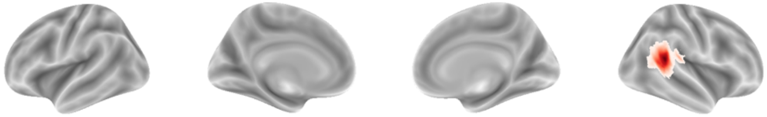

IC 12
(Most correlated with IC18 with r=0.96)

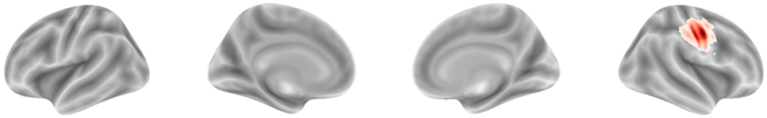

IC 13
(Most correlated with IC16 with r=0.97)

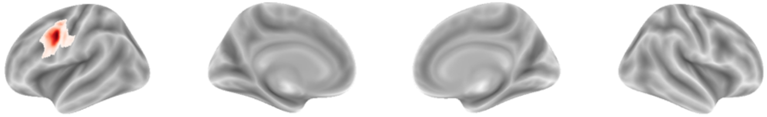

IC 14

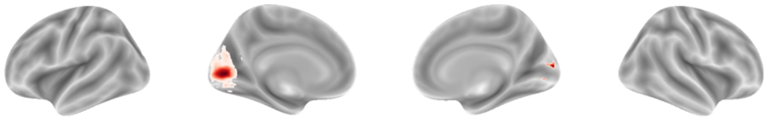

IC 15
(Most correlated with IC15 with r=0.95)

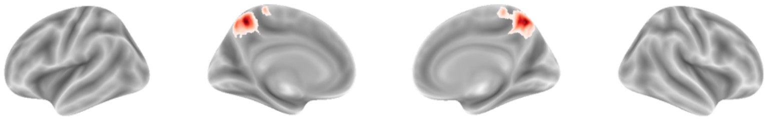

IC 16

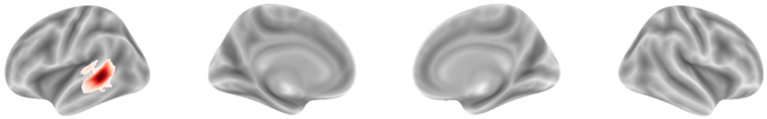

IC 17
(Most correlated with IC9 with r=0.79)

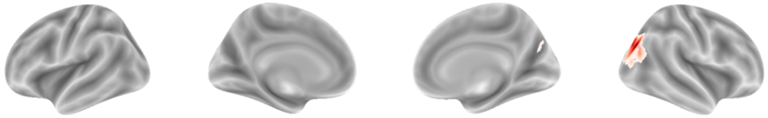

IC 18

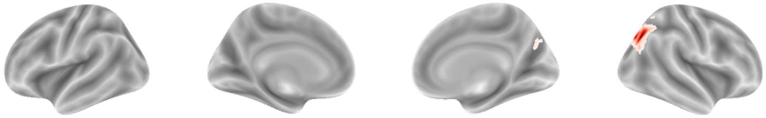

IC 19

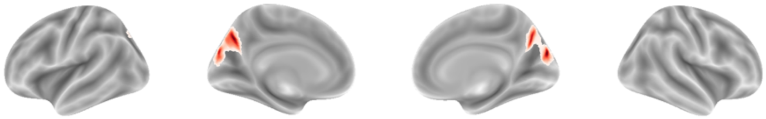

IC 21
(Most correlated with IC21 with r=0.87)

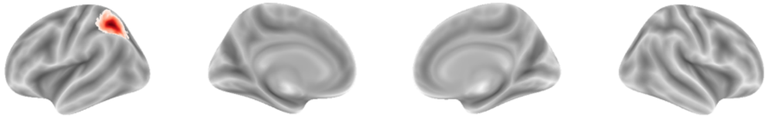

IC 22

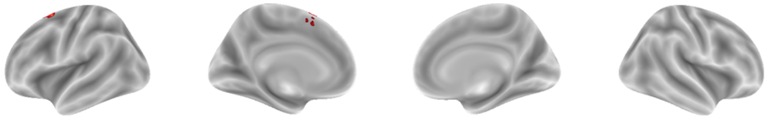

IC 23
(Most correlated with IC19 with r=0.90)

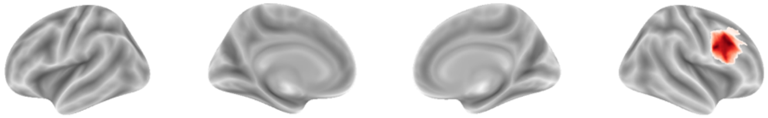

IC 24
(Most correlated with IC22 with r=0.94)

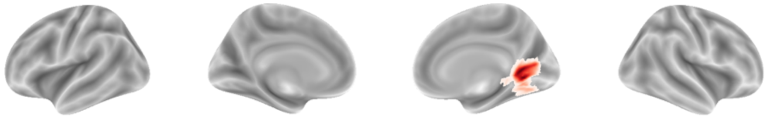

IC 25

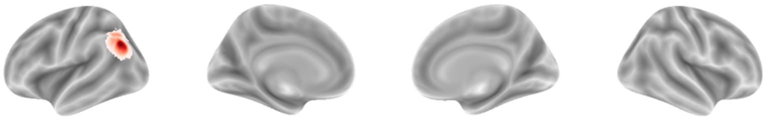

IC 26
(Most correlated with IC32 with r=0.92)

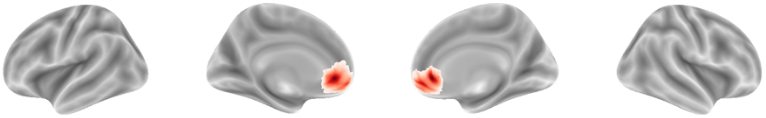

IC 27
(Most correlated with IC44 with r=0.87)

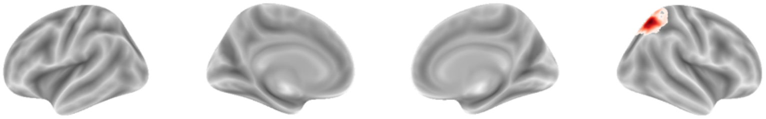

IC 28
(Most correlated with IC26 with r=0.85)

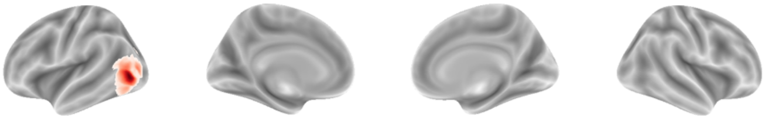

IC 29

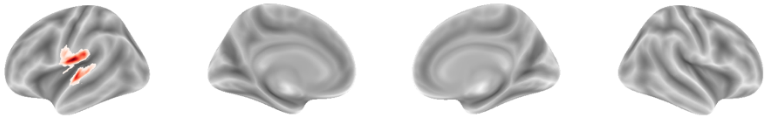

IC 30
(Most correlated with IC28 with r=0.88)

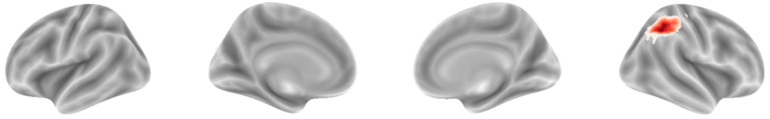

IC 31

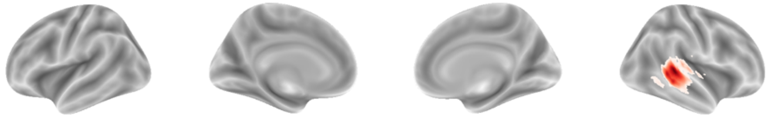

IC 32
(Most correlated with IC14 with r=0.70)

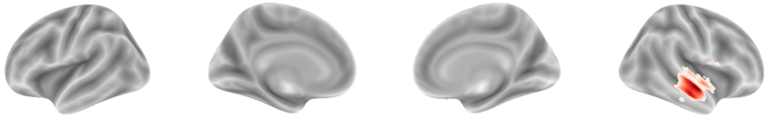

IC 33

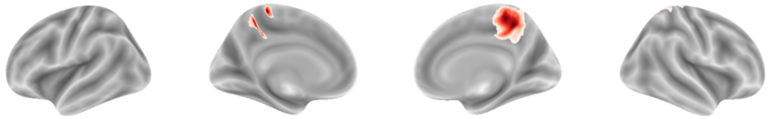

IC 34
(Most correlated with IC50 with r=0.96)

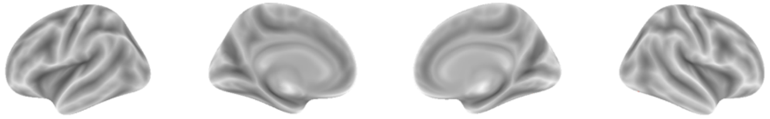

IC 35
(Most correlated with IC35 with r=0.86)

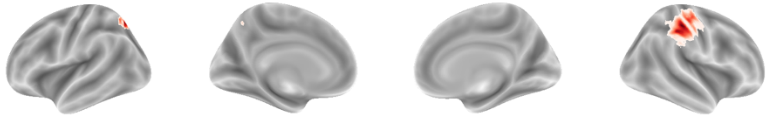

IC 36
(Most correlated with IC55 with r=0.91)

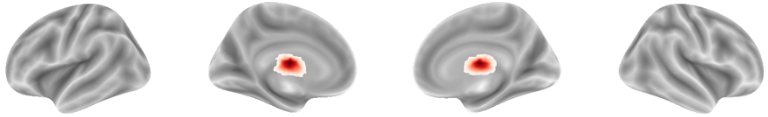

IC 37

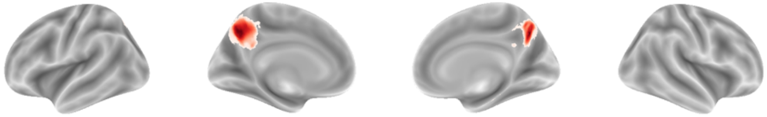

IC 38

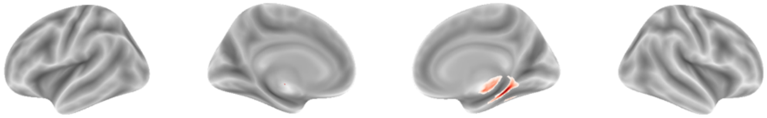

IC 39

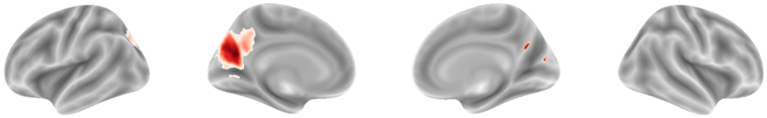

IC 40

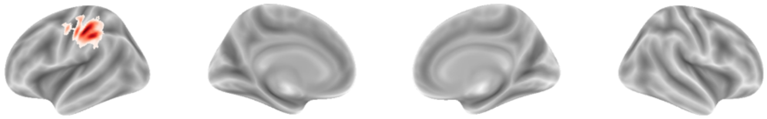

IC 41
(Most correlated with IC58 with r=0.86)

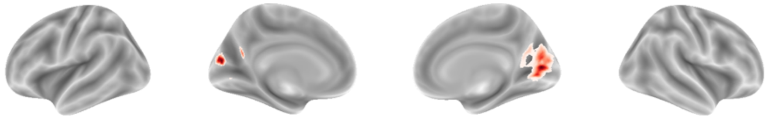

IC 42
(Most correlated with IC59 with r=0.92)

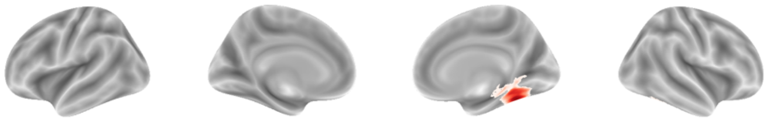

IC 43
(Most correlated with IC43 with r=0.92)

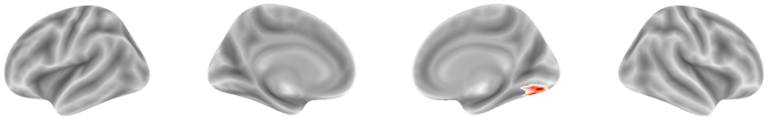

IC 44

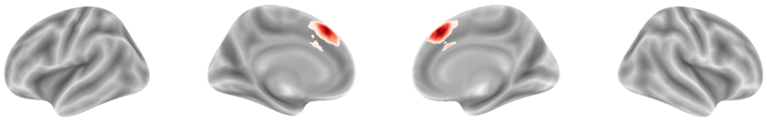

IC 45

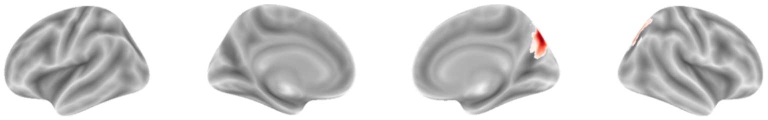

IC 46

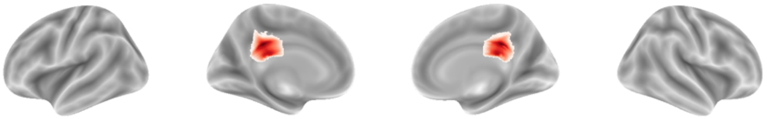

IC 47
(Most correlated with IC47 with r=0.92)

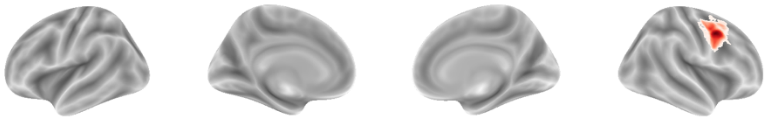

IC 48

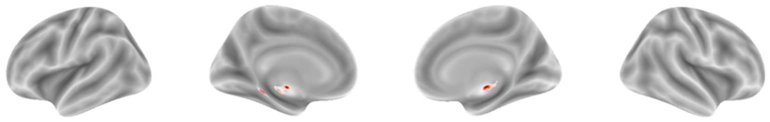

IC 49
(Most correlated with IC31 with r=0.88)

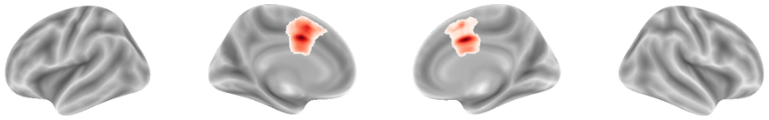

IC 50
(Most correlated with IC29 with r=0.84)

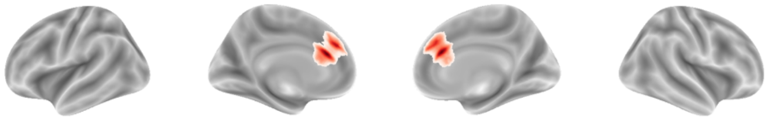

IC 51

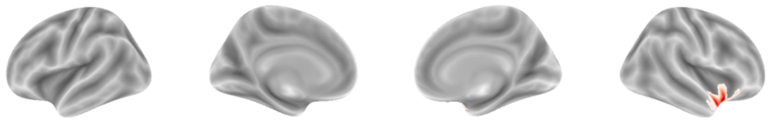

IC 52

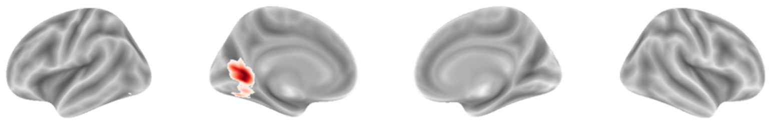

IC 53
(Most correlated with IC51 with r=0.84)

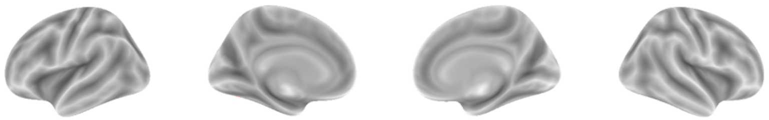

IC 54
(Most correlated with IC62 with r=0.77)

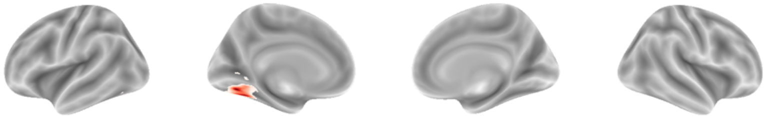

IC 55
(Most correlated with IC54 with r=0.91)

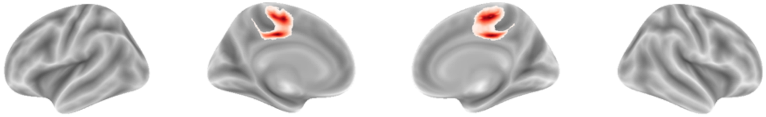

IC 56

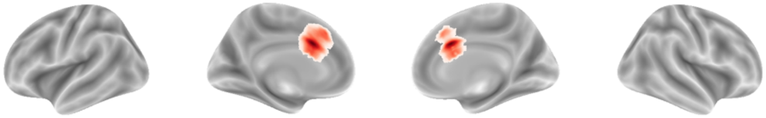

IC 57

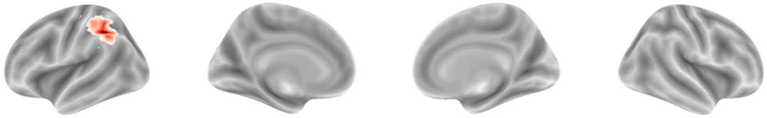

IC 58
(Most correlated with IC78 with r=0.97)

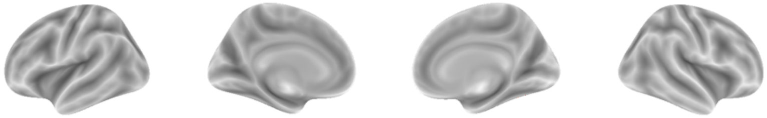

IC 59
(Most correlated with IC70 with r=0.59)

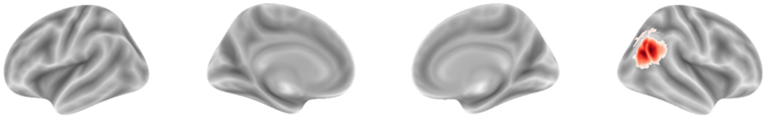

IC 60
(Most correlated with IC42 with r=0.89)

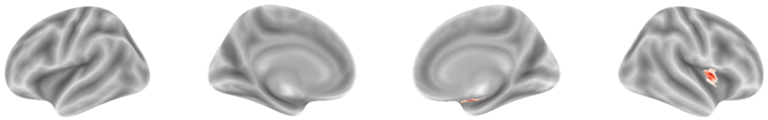

IC 61

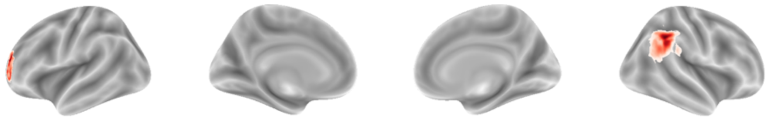

IC 62

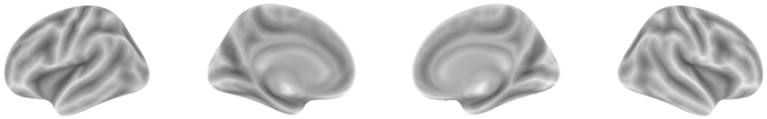

IC 63
(Most correlated with IC66 with r=0.88)

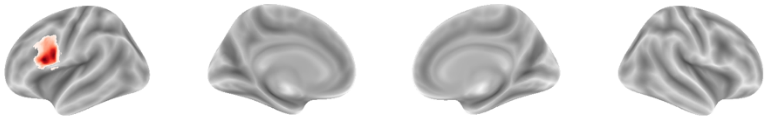

IC 64

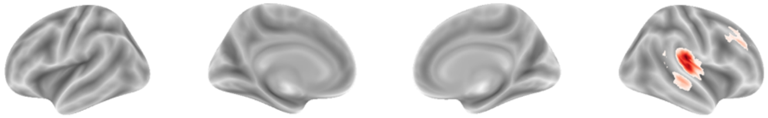

IC 65
(Most correlated with IC68 with r=0.88)

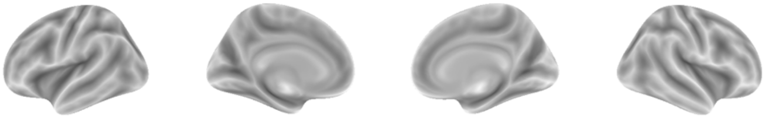

IC 66

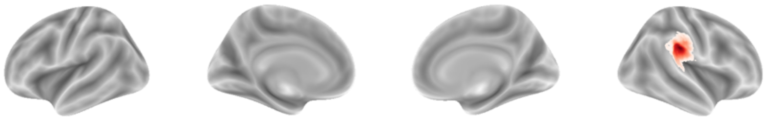

IC 67
(Most correlated with IC40 with r=0.78)

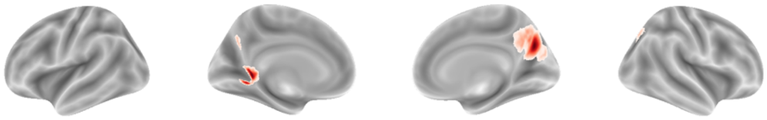

IC 68
(Most correlated with IC60 with r=0.88)

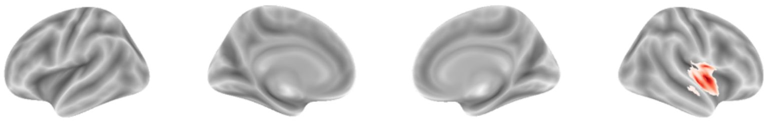

IC 69

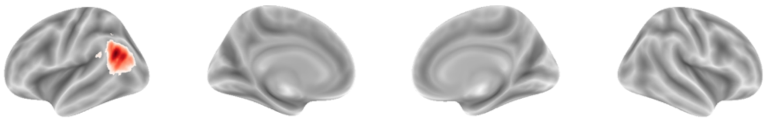

IC 70

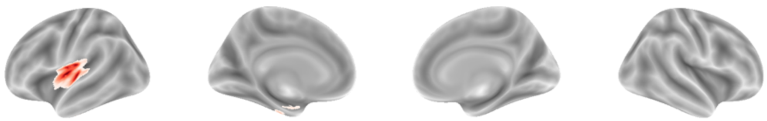

IC 71

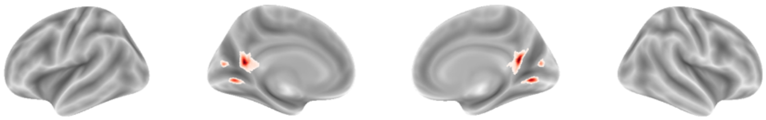

IC 72

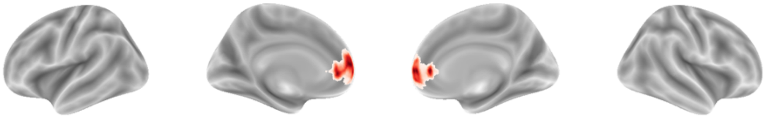

IC 73

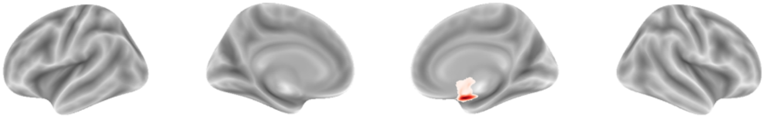

IC 74

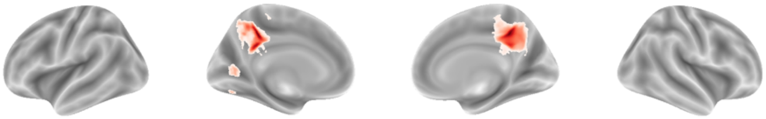

IC 75

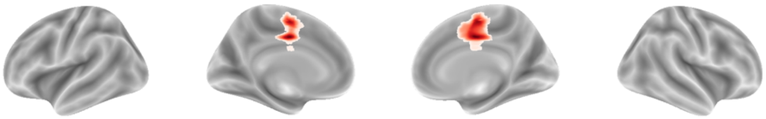

IC 76
(Most correlated with IC63 with r=0.79)

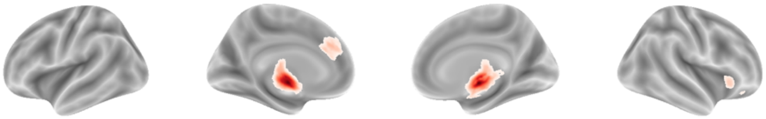

IC 77
(Most correlated with IC57 with r=0.84)

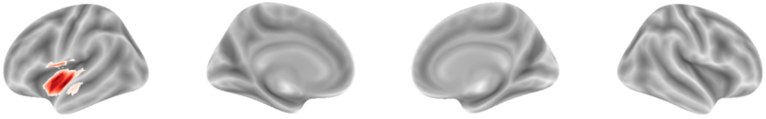

IC 78

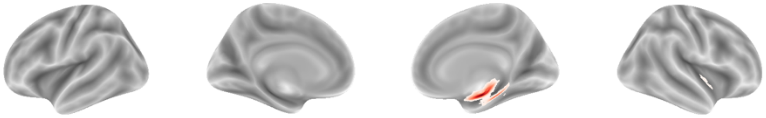

IC 79

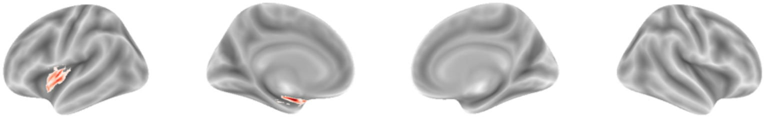

IC 80
(Most correlated with IC86 with r=0.78)

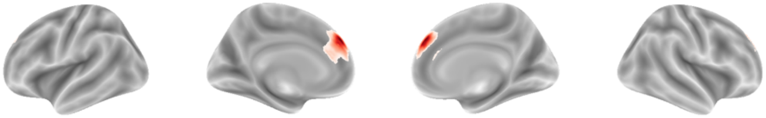

IC 81

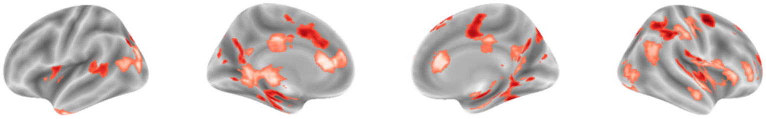

IC 82

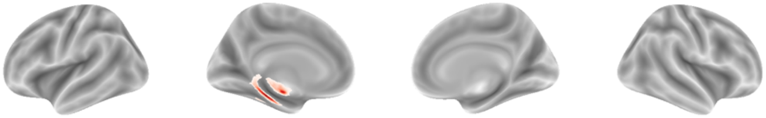

IC 83

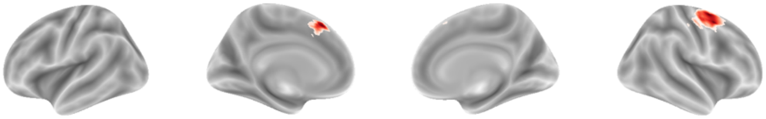

IC 84
(Most correlated with IC67 with r=0.82)

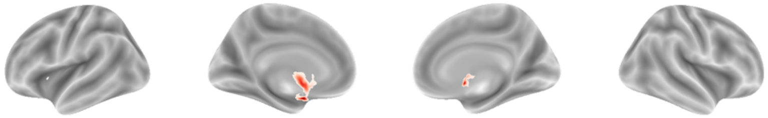

IC 85

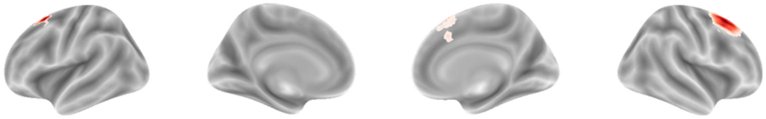

IC 86

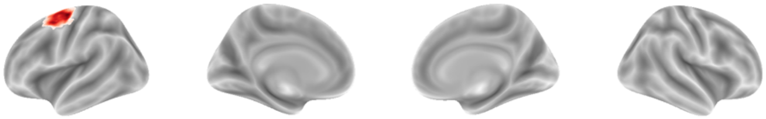

IC 87

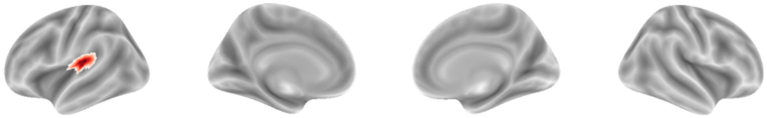

IC 88

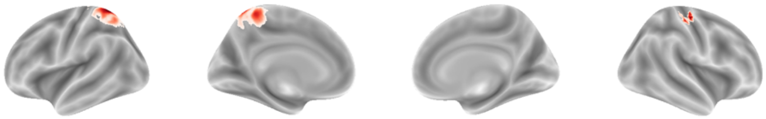

IC 89

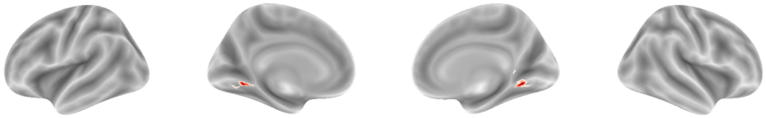

IC 90

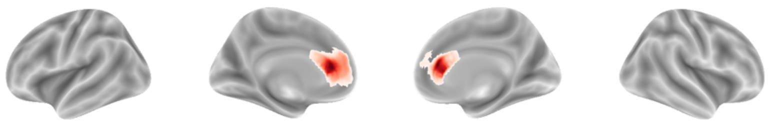

IC 91
(Most correlated with IC85 with r=0.87)

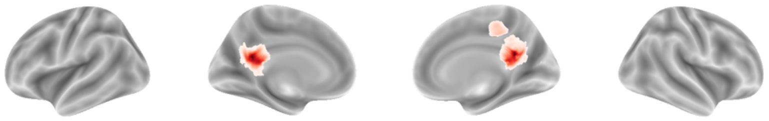

IC 92
(Most correlated with IC72 with r=0.83)

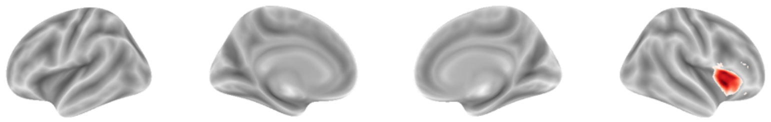

IC 93
(Most correlated with IC76 with r=0.81)

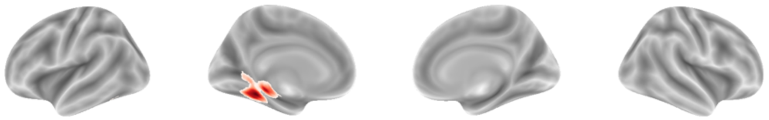

IC 94
(Most correlated with IC80 with r=0.85)

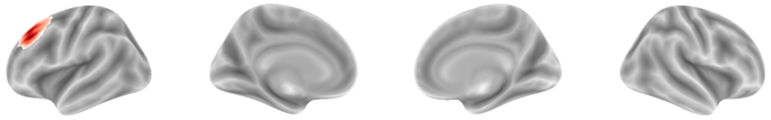

IC 95

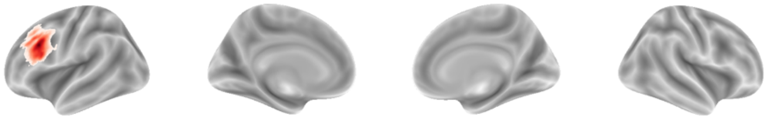

IC 96

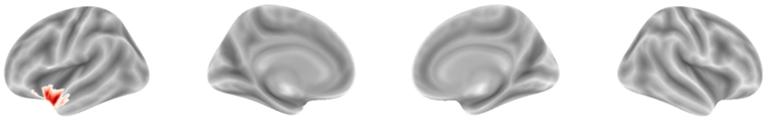

IC 97

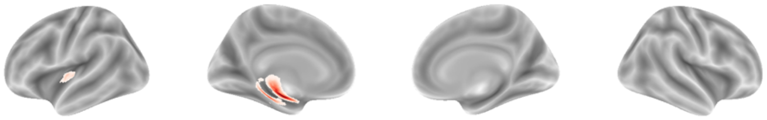

IC 98

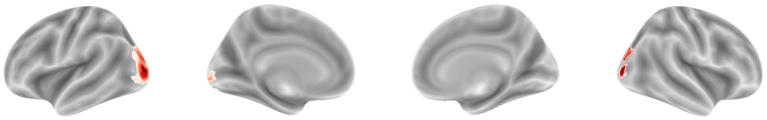

IC 99

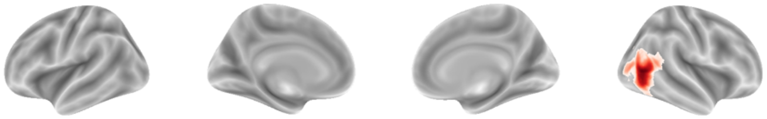

IC 100

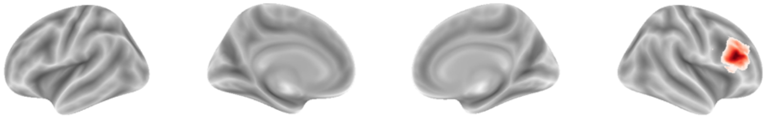

IC 101

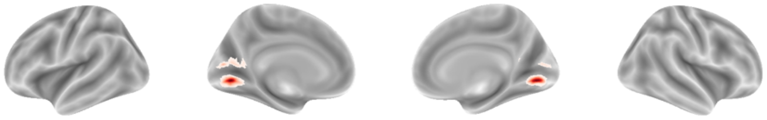

IC 102

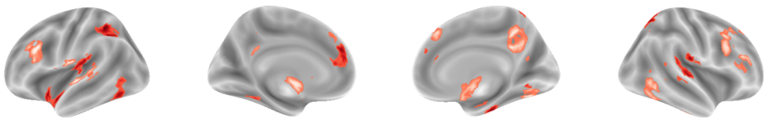

IC 103

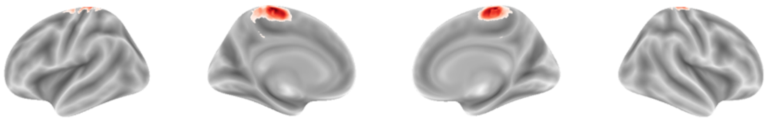

IC 104

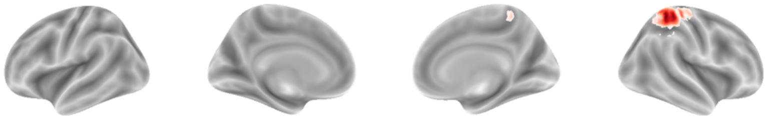

IC 105

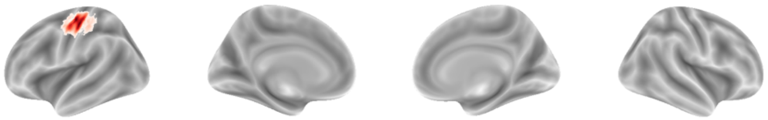

IC 106

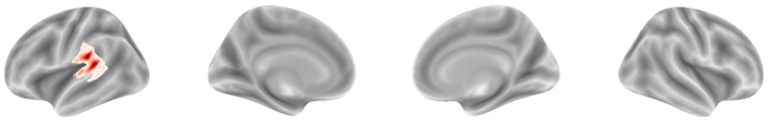

IC 107
(Most correlated with IC64 with r=0.64)

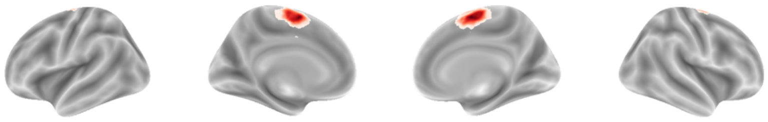

IC 108
(Most correlated with IC96 with r=0.70)

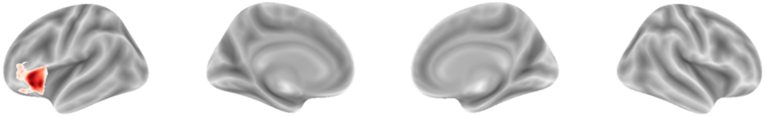

IC 109
(Most correlated with IC90 with r=0.68)

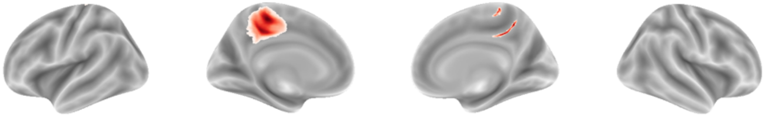

IC 110

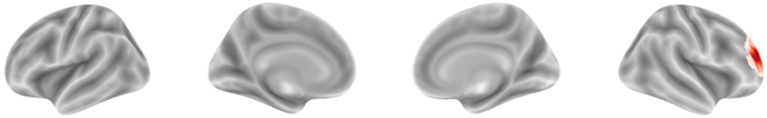

IC 111

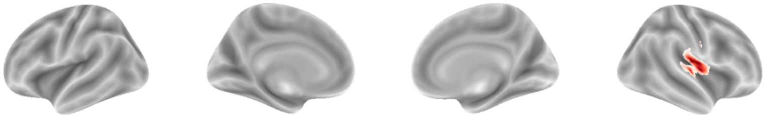

IC 112
(Most correlated with IC88 with r=0.47)

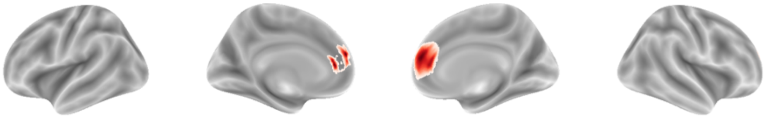

IC 113

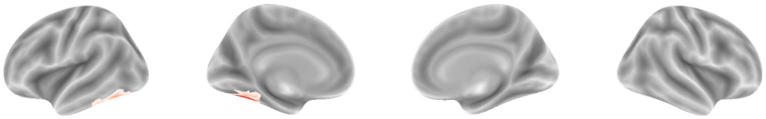

IC 114
(Most correlated with IC99 with r=0.48)

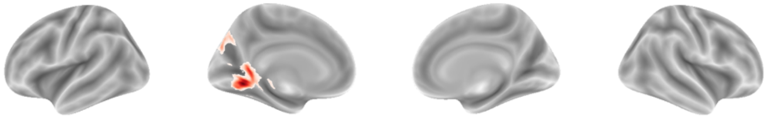

IC 115

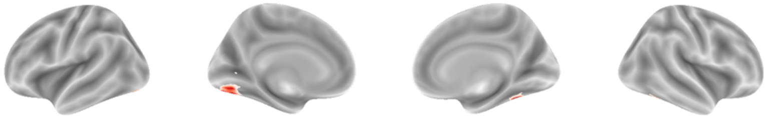

IC 116

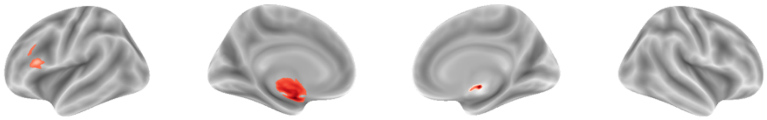

IC 117

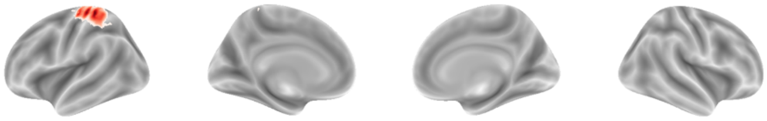

IC 118

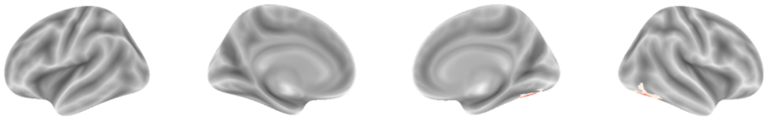

IC 119

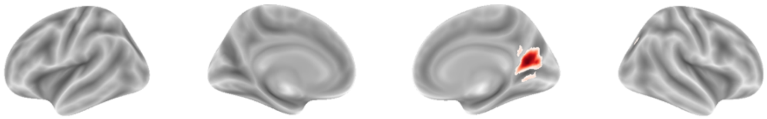

IC 120

(Most correlated with IC87 with r=0.75)

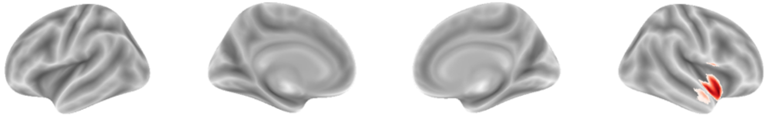

IC 121

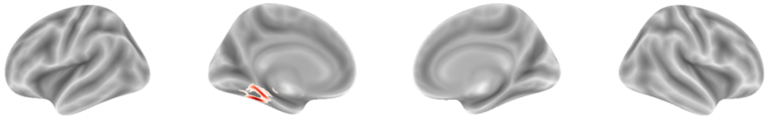

IC 122

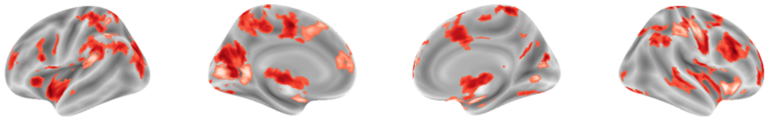

IC 123

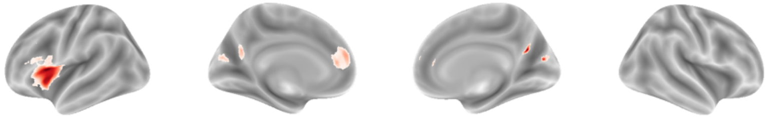

IC 124

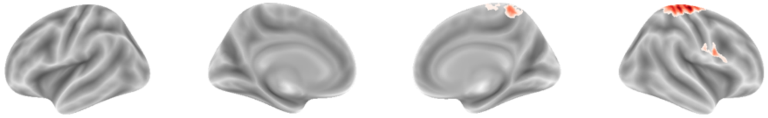

IC 125

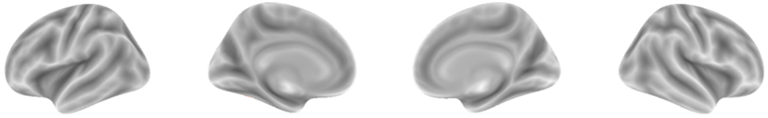

IC 126

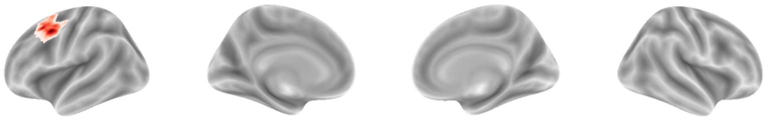

IC 127

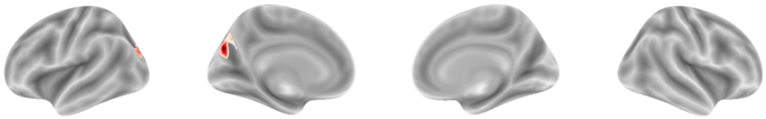

IC 128

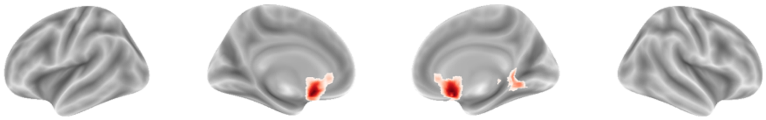

IC 129

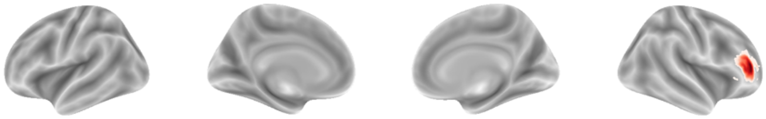

IC 130

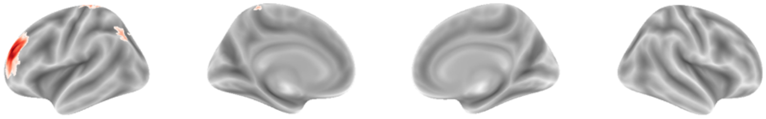

IC 131

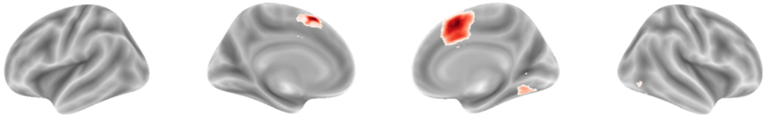

IC 132

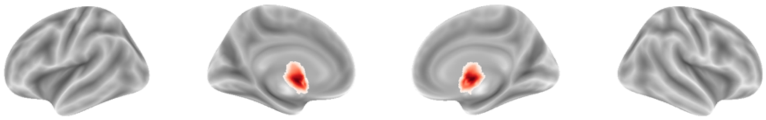

IC 133

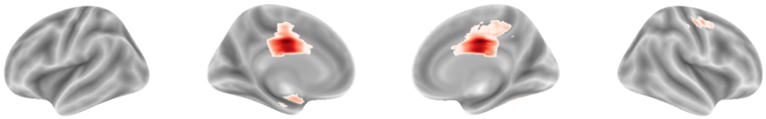

IC 134

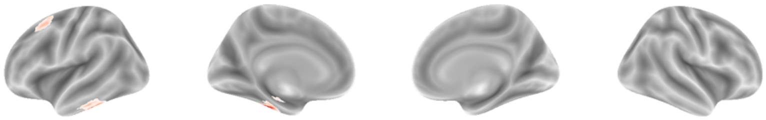

IC 135

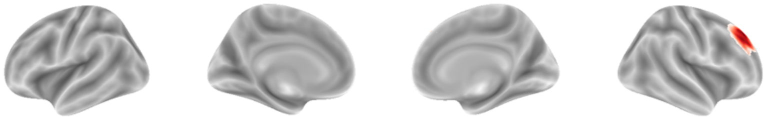

IC 136

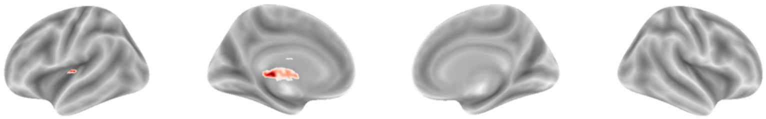

IC 137

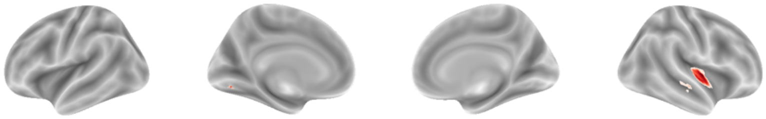

IC 138

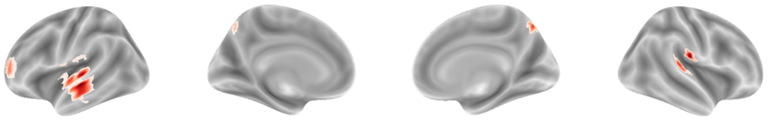

IC 139

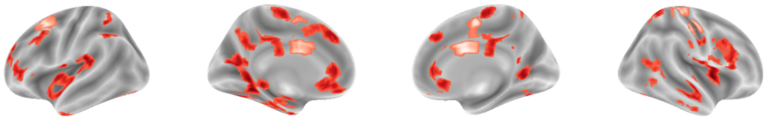

IC 140

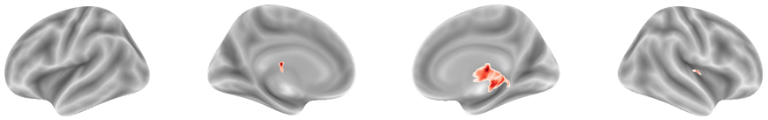

IC 141

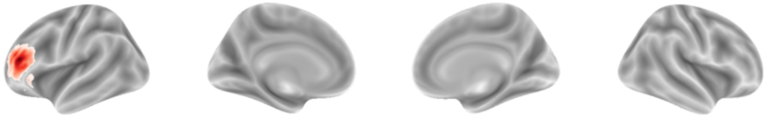

IC 142

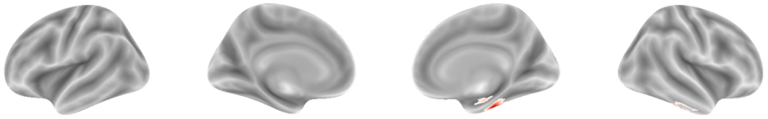

IC 143

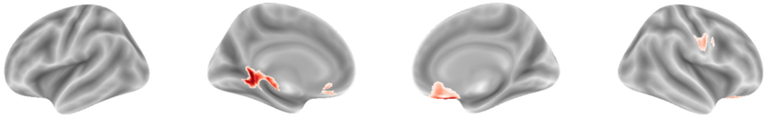

IC 144

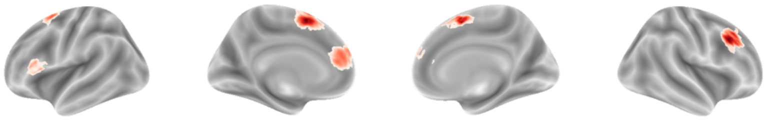

IC 145

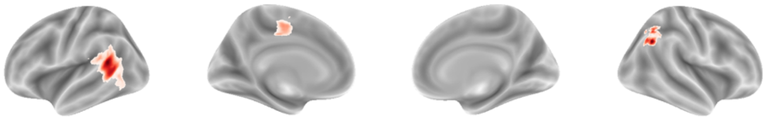

IC 146

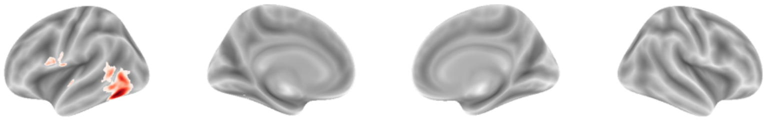

IC 147

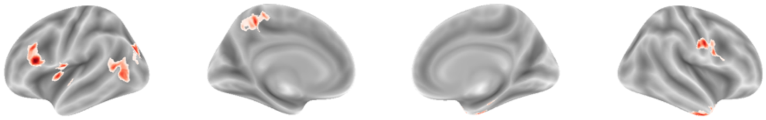

IC 148

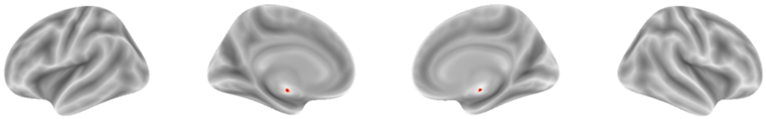

IC 149

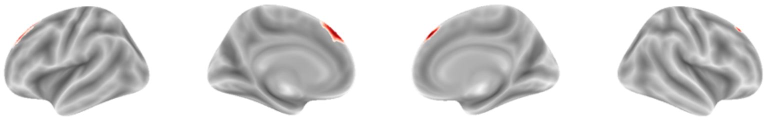

IC 150

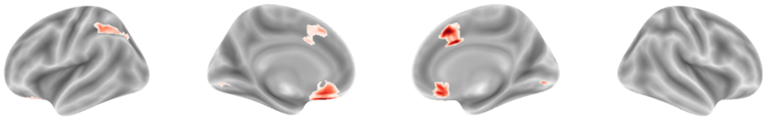

Figure S4. 50-dimensional dendrogram. *Dendrogram generated using hierarchical clustering of the IC time courses with 50 dimensions. A red border highlights ICs that show the highest spatial correlation with the 49 significant ICs identified from the 100-dimensional analysis in the Mantel test. The corresponding IC number from the 100-dimensional analysis is labeled below each highlighted IC.*

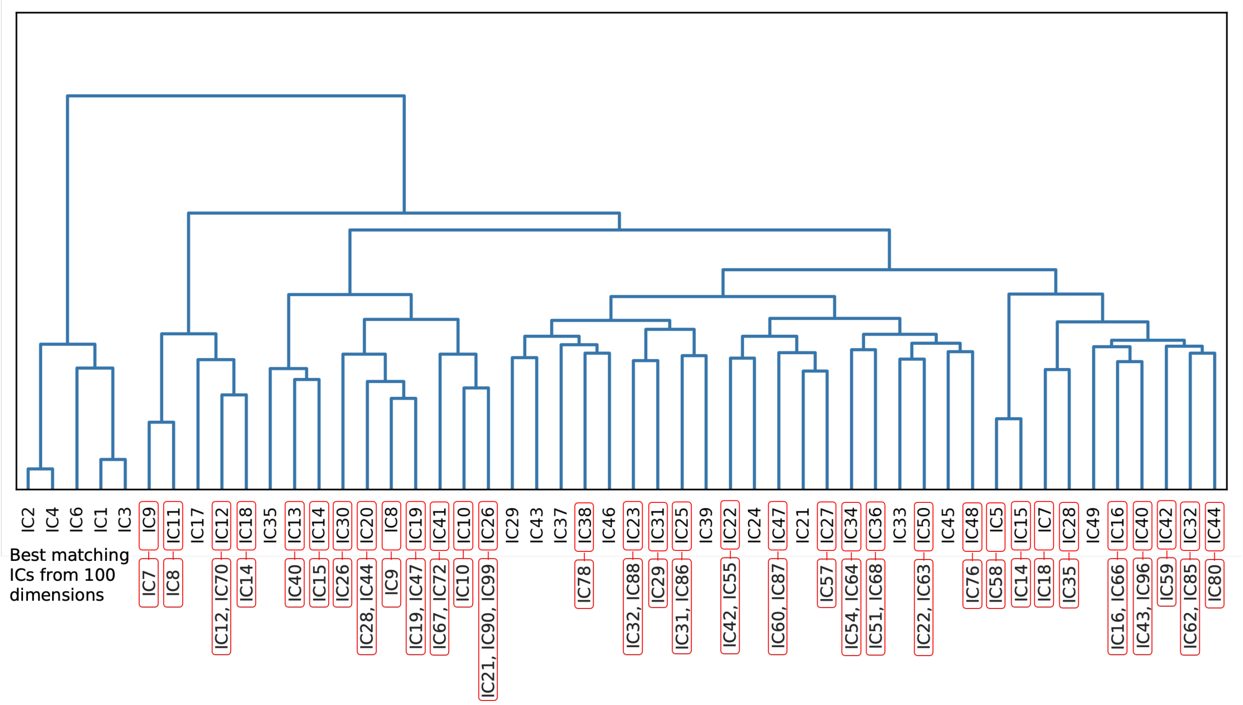

Figure S5. **100-dimensional dendrogram.** *Dendrogram generated using hierarchical clustering of the IC time courses with 100 dimensions.*

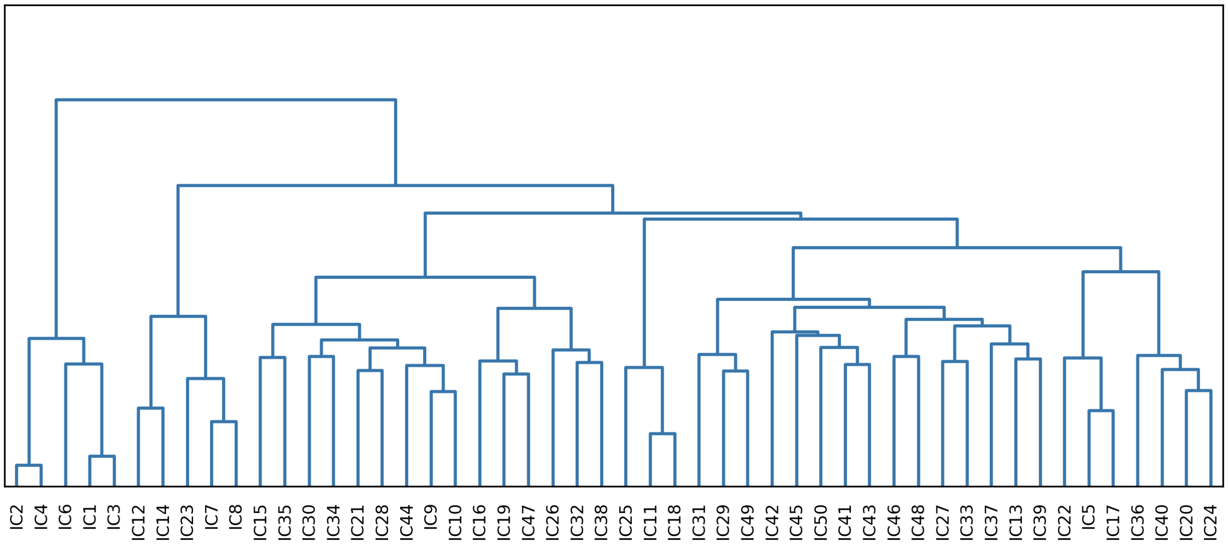

Figure S6. 150-dimensional dendrogram. *Dendrogram generated using hierarchical clustering of the IC time courses with 150 dimensions. A red border highlights ICs that show the highest spatial correlation with the 49 significant ICs identified from the 100-dimensional analysis in the Mantel test. The corresponding IC number from the 100-dimensional analysis is labeled below each highlighted IC.*

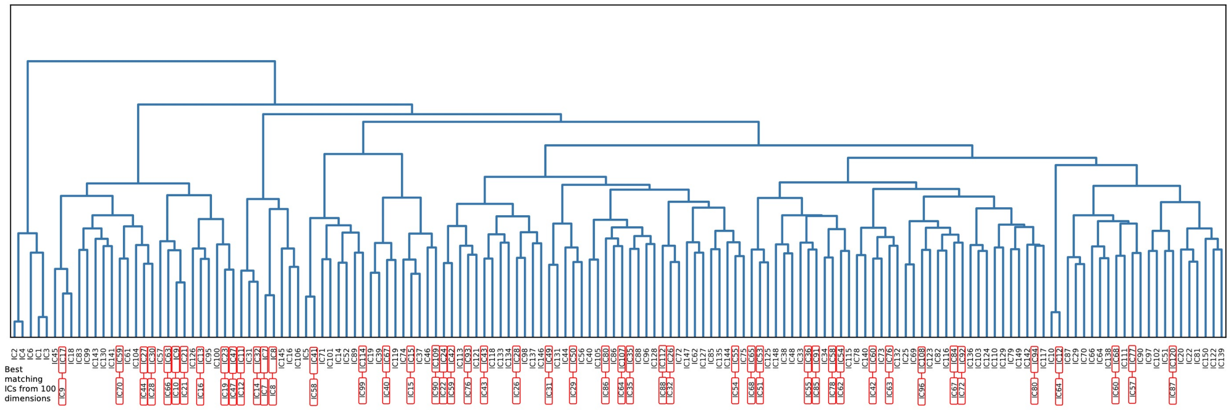

Table S1. Network Information. *Each row is the peak z-score voxel from statistically significant false discovery rate (FDR) corrected wellbeing-modulated independent component (IC) networks, as determined by a Mantel test (all FDR-corrected ps < 0.01). Rows are colour coded by co-activated networks or clades (see Fig. 1 and 3-5). The number of voxels is for the whole IC network (which might consist of multiple other regions). 'Decoding' provides the top 10 term-based meta-analysis associations at both the peak Montreal Neurological Institute (MNI) coordinate and whole clade for only cognitive/behavioural related terms (excluding, e.g., anatomical terms). Other abbreviations: A/P = anterior/posterior; I/S = inferior/superior; L/R = left/right; un/corr = un/corrected.*

				Peak MNI Coordinates			Decoding (N=10)						
**Clade**	**IC**	**Region**	**Voxels**	**x (L/R)**	**y (A/P)**	**z (I/S)**	**Coordinate (z > 0)**	**Clade**	**ρ**	**Peak time**	**Z thresh**	**Uncorr p**	**FDR corr p**
1	29	Perigenual Anterior Cingulate Cortex	241	-2	28	29	Pain, painful, gain, noxious, monetary, mood, nociceptive	Pain, painful, conflict, referential, self referential, ptsd, rehearsal, coordination, motor performance, default	0.2793	5451	0.4	0.0003	0.0019
1	31	Anterior Midcingulate Cortex	100	2	3	44	Pain, finger, movement, autonomic, movements, sensations, execution, tapping, index finger, target detection		0.2920	612	0.6	0.0004	0.0021
1	32	Ventromedial Prefrontal Cortex	90	-2	45	-3	Default mode, default, mood, reward, valence, smoking, money, gambling, monetary, value		0.3107	2719	0.6	0.0022	0.0054
1	43	Right Cerebellum	184	23	-75	-24	Interpersonal		0.2991	2241	0.3	0.0024	0.0056
1	50	Right Cerebellum	86	26	-74	-45	Verbal working		0.3921	3411	0.6	0.0001	0.0009
1	51	Left Cerebellum	74	-35	-68	-49	Cognitive functions, suppressed, working memory, working		0.2675	2788	0.7	0.0004	0.0021
1	54	Posterior Middle Cingulate Cortex	309	-2	-26	49	Foot, confidence, coordination		0.2557	4519	0.3	0.0010	0.0036
1	60	Right Posterior Insula	143	44	-11	4	Pain, auditory, music, discriminative, painful, musical, nociceptive, listening, audiovisual, noxious		0.3845	2445	0.5	0.0001	0.0009
1	64	Dorsal Medial Frontal Cortex	270	2	-5	63	Movements, movement, motor imagery, hand, sensorimotor, motor task, execution, finger, finger movements, imagery		0.2334	2598	0.4	0.0032	0.0066
1	68	Left Cerebellum	273	-20	-77	-46	Stimulation, pain, electrical, sensory, navigation, sensorimotor		0.2934	4314	0.3	0.0009	0.0036
1	78	Right Cerebellum	460	35	-64	-48	Loop		0.2326	2782	0.2	0.0018	0.0046
1	80	Left Superior Frontal Sulcus	320	-26	27	46	Default, default network, autobiographical, default mode, real world, conceptual, episodic, autobiographical memory, memory, episodic memory		0.2167	2479	0.3	0.0044	0.0082
1	86	Dorsal Medial Frontal Cortex	146	2	43	43	Inference, mind tom, arousal, emotional, social, tom		0.2574	5259	0.5	0.0001	0.0009
1	87	Right Anterior Insula	827	44	-1	-9	Pain, intensity, aversive, taste, painful, articulatory, multisensory		0.2148	263	0.2	0.0027	0.0060
1	88	Perigenual Anterior Cingulate Cortex	61	8	47	14	Default, beliefs, default mode		0.3378	1000	0.7	0.0001	0.0009
2	42	Right Putamen	1374	23	-8	1	Finger movements, finger tapping, movements, finger, movement, tapping,	Gain,	0.2700	3852	0.1	0.0012	0.0036

							sensorimotor, handed, sexual, execution	pain, reward, anticipation, painful, stop, monetary reward, monetary, sexual, mood					
2	55	Thalamus	654	2	-12	14	Monetary reward, monetary, anticipation, retrieved, video clips, self reported		0.3812	391	0.1	0.0011	0.0036
2	57	Left Posterior Insula	346	-44	-7	-6	Experiences, pain, unfamiliar, acoustic		0.2478	51	0.3	0.0001	0.0009
2	63	Ventral Tegmental Area	274	-2	-17	-6	Reward, motivational, rewards, reinforcement learning, behaviours, reinforcement, motivation, addiction, choices, monetary		0.3229	1527	0.3	0.0003	0.0019
2	67	Left Pallidum	97	-17	-1	-2	Movement, movements, execution, coordination, tapping		0.2518	2198	0.6	0.0056	0.0098
2	72	Right Anterior Insula	1061	38	23	3	Task, gain, calculation, mood, pain, choose, german, demands, tasks, orthographic		0.3142	4697	0.2	0.0004	0.0021
2	96	Left Anterior Insula	359	-35	23	3	Gain, phonological, language, orthographic, competing, words, heard, demands, pain		0.2432	1966	0.3	0.0025	0.0057
3	7	Right Heschl's gyrus	96	59	-11	11	Auditory, pitch, sounds, listening, touch, listened, noise, tactile, production, sound	Auditory, listening, speech, sounds, acoustic, sound, pitch, listened, speech perception, audiovisual	0.3709	5111	0.3	0.0014	0.0039
3	8	Left Supramarginal Gyrus	418	-50	-46	25	Mind tom, tom, phonological, social interaction, reading, speech production, language, listening, linguistic, theory mind		0.5245	1203	0.1	0.0001	0.0009
3	12	Right Superior Temporal Gyrus	176	53	-46	16	Theory mind, tom, mental states, mind tom, mind, motion, theory, watching, empathy, empathic		0.3690	2502	0.4	0.0012	0.0036
3	14	Right Superior Temporal Gyrus	281	53	-23	-3	Listening, speech, auditory, language, sentences, comprehension, voice, linguistic, sentence, speech perception		0.3508	1223	0.5	0.0011	0.0036
4	18	Right Precentral Gyrus	459	47	-16	40	Sensorimotor, finger, speech production, vocal, production	Speech production, production, oral, sensorimotor, vocal, naming, articulatory, movement, movements, finger	0.3753	9	0.2	0.0030	0.0063
5	15	Left Precuneus	241	2	-63	54	Spatial, calculation, judgement task,	Locations,	0.3413	836	0.3	0.0020	0.0050

							attention network, navigation	navigation, orienting, spatial attention, default, default mode, spatial, virtual, visual attention, location					
5	40	Left Precuneus	619	14	-65	31	Retrieval, episodic, episodic memory, memory, recognition memory, memory retrieval, amnestic, items, autobiographical		0.3578	1641	0.2	0.0011	0.0036
6	22	Right Lingual Gyrus	475	20	-60	2	Implicit	Place, navigation, objects, visual, episodic, encoding, visual stream, episodic memory, object, autobiographical	0.2430	3965	0.2	0.0029	0.0063
6	58	Right Calcarine Sulcus	55	14	-77	14	Sighted, visual, eye, eye fields, frontal eye, ageing, saccades, lexical		0.3463	1572	0.6	0.0007	0.0031
6	59	Right Cerebellum	179	32	-53	-26	Motor performance, deficient, sensorimotor, motor imagery, execution, movement, movements, hand		0.2318	5230	0.5	0.0035	0.0068
6	62	Left Cerebellum	313	-23	-69	-20	Movements, movement, production, sensorimotor, finger tapping, pitch, tapping, rhythm, musicians, execution		0.2367	2653	0.3	0.0034	0.0068
6	76	Left Parahippocampal Gyrus	229	-29	-41	-11	Episodic, navigation, episodic memory, autobiographical, memory, retrieval, encoding, objects, place, construction		0.3027	1975	0.5	0.0035	0.0068
6	85	Posterior Cingulate Cortex	1279	-2	-52	15	Default, default mode, autobiographical, episodic, memory retrieval, semantic memory, recollection, retrieval, default network, dmn		0.2723	3192	0.1	0.0040	0.0076
6	99	Left Lingual Gyrus	287	-11	-54	-5	Picture, navigation, words, semantic, sensorimotor		0.2383	1838	0.3	0.0014	0.0039
7	9	Right Intraparietal Sulcus	314	35	-74	30	Spatial, object, eye fields, virtual, objects, navigation, tasks, frontal eye, visuospatial, rotation	Tasks, working memory, working, task, visuospatial, attention, calculation, visual, spatial, attentional	0.3618	2219	0.3	0.0053	0.0095
7	10	Left Intraparietal Sulcus	55	-32	-77	33	Retrieval, memory, encoded, navigation, retrieved, episodic, object, episodic memory, ageing, visual		0.3372	3422	0.7	0.0001	0.0009
7	16	Left Premotor Cortex	270	-47	-3	41	Working memory, working, speech production, saccades, phonological, production, eye, sensorimotor, rehearsal, language		0.5137	4027	0.2	0.0001	0.0009
7	19	Right Dorsolateral Prefrontal Cortex	127	44	12	32	Mood, task, interference, demands, cognitive control, tasks,		0.3320	4498	0.7	0.0012	0.0036

							stroop, response time, stroop task, ptsd						
7	21	Left Intraparietal Sulcus	281	-35	-56	48	Memory, working memory, working, retrieval, recognition memory, word, calculation, memory retrieval, words, retrieved		0.2815	3645	0.4	0.0023	0.0055
7	26	Bilateral Middle Temporal Visual Motion Area	307	-41	-76	-3	Visual, objects, visual motion, motion, face recognition, object, face, negative neutral, orthographical, stream		0.3630	2096	0.2	0.0015	0.0041
7	28	Right Intraparietal Sulcus	622	38	-53	45	Tasks, working memory, working, task, memory, load, attentional, calculation, maintenance, demand		0.2677	1987	0.2	0.0048	0.0088
7	35	Right Intraparietal Sulcus	594	44	-32	49	Finger, tactile, planning, movements, hand, spatial, finger movements, tasks, sensorimotor, sequential		0.3522	879	0.3	0.0001	0.0009
7	44	Right Intraparietal Sulcus	153	23	-60	54	Eye movements, eye, visual, eye fields, attention network, spatial, movements, objects, attention, visually		0.2708	2384	0.4	0.0008	0.0034
7	47	Right Frontal Eye Field	55	41	2	47	Eye fields, frontal eye, eye, spatial attention, eye movements, action, eye field, orienting, saccades, tasks		0.2371	481	0.7	0.0005	0.0023
7	66	Left Dorsolateral Prefrontal Gyrus	501	-50	10	25	Phonological, language, semantic, orthographic, words, languages, word, linguistic, reading, tasks		0.3355	801	0.2	0.0002	0.0015
7	70	Middle Temporal Visual Motion Area	235	44	-64	11	Motion, video clips, video, action observation, hands, actions, body, eye, eye movements, visual		0.2994	3546	0.5	0.0002	0.0015
7	90	Midcingulate Cortex	1008	-11	-41	48	Self referential, referential, video clips		0.2271	519	0.2	0.0005	0.0023
